# A descriptive analysis of the Spatio-temporal distribution of intestinal infectious diseases in China

**DOI:** 10.1186/s12879-019-4400-x

**Published:** 2019-09-02

**Authors:** Ying Mao, Ning Zhang, Bin Zhu, Jinlin Liu, Rongxin He

**Affiliations:** 1 School of Public Policy and Administration, Xi’an Jiaotong University, Xi’an, 710049 China; 2 Department of Public Policy, City University of Hong Kong, Hong Kong, 999077 China

**Keywords:** Spatial epidemiology, Seasonal trend, Long-term trend, Intestinal infectious diseases, Moran’s I, China

## Abstract

**Background:**

Intestinal infectious diseases (IIDs) have caused numerous deaths worldwide, particularly among children. In China, eight IIDs are listed as notifiable infectious diseases, including cholera, poliomyelitis, dysentery, typhoid and paratyphoid (TAP), viral Hepatitis A, viral Hepatitis E, hand-foot-mouth disease (HFMD) and other infectious diarrhoeal diseases (OIDDs). The aim of the study is to analyse the spatio-temporal distribution of IIDs from 2006 to 2016.

**Methods:**

Data on the incidence of IIDs from 2006 to 2016 were collected from the public health science data centre issued by the Chinese Center for Disease Control and Prevention. This study applied seasonal decomposition analysis, spatial autocorrelation analysis and space-time scan analysis. Plots and maps were constructed to visualize the spatio-temporal distribution of IIDs.

**Results:**

Regarding temporal analysis, the incidence of HFMD and Hepatitis E showed a distinct increasing trend, while the incidence of TAP, dysentery, and Hepatitis A presented decreasing trends over the last decade. The incidence of OIID remained steady. Summer is the season with the greatest number of cases of different IIDs. Regarding the spatial distribution, approximately all *p* values for the global Moran’s I from 2006 to 2016 were less than 0.05, indicating that the incidences of the epidemics were unevenly distributed throughout the country. The high-risk areas for HFMD and OIDD were located in the Beijing-Tianjin-Tangshan (BTT) region and south China. The high-risk areas for TAP were located in some parts of southwest China. A higher incidence rates for dysentery and Hepatitis A were observed in the BTT region and some west provincial units. The high-risk areas for Hepatitis E were the BTT region and the Yangtze River Delta area.

**Conclusions:**

Based on our temporal and spatial analysis of IIDs, we identified the high-risk periods and clusters of regions for the diseases. HFMD and OIDD exhibited high incidence rates, which reflected the negligence of Class C diseases by the government. At the same time, the incidence rate of Hepatitis E gradually surpassed Hepatitis A. The authorities should pay more attention to Class C diseases and Hepatitis E. Regardless of the various distribution patterns of IIDs, disease-specific, location-specific, and disease-combined interventions should be established.

**Electronic supplementary material:**

The online version of this article (10.1186/s12879-019-4400-x) contains supplementary material, which is available to authorized users.

## Background

Intestinal infectious diseases (IIDs), also known as infectious enteric diseases, are transmitted via the faecal-oral route through contaminated food, water, or fomites. The main symptoms of intestinal infectious diseases include nausea, vomiting, fever, headache, limb pain, abdominal pain, loss of appetite, systemic poisoning, diarrhoea, and other gastrointestinal symptoms, which may lead to death if not treated promptly. IIDs have posed public health threats and resulted in severe social and economic burdens due to the high incidence and morbidity rates, particularly in young children [1–3]. Several large, severe global disease outbreaks have been associated with IIDs, such as the outbreaks of HFMD in east and southeast Asia in the early twenty-first century [4], the constantly high burden of cholera that persists in many African countries, and the cholera outbreaks with active cholera transmission in many sub-Saharan African countries [5]. According to the sustainable development goals (SDG), we should combat hepatitis, water-borne diseases and other communicable diseases. IIDs represent a significant obstacle to achieving SDG [6].

The Chinese government enacted the *Law of the People’s Republic of China on the Prevention and Treatment of Infectious Diseases* and regularly reports cases of notifiable diseases to effectively monitor and control infectious diseases. All reported infectious diseases are divided into Classes A, B and C in terms of severity, which is shown in Additional file 1. In China, IIDs are one of the main types of infectious diseases, presenting the characters of a high morbidity rate and low mortality rate [7]. Eight IIDs are listed in the law, including cholera, poliomyelitis, dysentery, typhoid and paratyphoid (TAP), viral Hepatitis A, viral Hepatitis E, hand-foot-mouth disease (HFMD) and other infectious intestinal diseases (OIIDs). Among these diseases, cholera and poliomyelitis have been almost completely eradicated. In contrast, according to the data reported by the Chinese CDC in 2017, viral Hepatitis A, viral Hepatitis E, and dysentery are among the top five most severe diseases in Class B. At the same time, HFMD and OIDDs were the most severe diseases in class C. The status of intestinal infectious diseases requires further attention.

Previous studies have investigated the epidemiology of different intestinal infectious diseases. First, temporal analysis of different intestinal infectious diseases was conducted. The initial efforts in conducting a temporal analysis investigated the temporal variations in the incidence rates of different diseases in different age groups and areas. For example, Tian et al. analysed the temporal characteristics of HFMD and the relationship between meteorological factors and the incidence of HFMD in Beijing, China. May to July is the period with peak HFMD incidence each year in Beijing [8]. Xiao et al. compared the temporal trends for two types of HFMD cases, namely, the daily series of disease counts of mild and severe HFMD cases reported in mainland China in the period from 2009 to 2014 [9]. Yang et al. applied the Grey Model First Order One Variable [GM (1, 1)] to predict the trend in the incidence of typhoid using data collected from Wuhan City in China from 2004 to 2015 [10]. Liu et al. forecasted the incidence of bacillary dysentery diseases by applying the seasonal trend model based on the moving average method, which forecast the incidence of Hepatitis A in 2015 [11]. Ming et al. investigated the seasonal signals and long-term trends in a series of 10 IGS sites in China [12]. Xing et al. conducted a study examining the epidemiological characteristics in China from 2008 to 2012, emphasizing seasonal patterns among people at different ages [13]. Temporal analyses of infectious diseases have allowed researchers to develop a wide range of research methods, namely, time series analysis and seasonal analysis. However, temporal analysis failed to reflect the overall circumstances of infectious intestinal diseases.

On the other hand, the academic world gradually began to realize the importance of performing spatial analysis of infectious diseases. Zhang et al. conducted a space-time scan analysis of HFMD in Liaocheng City in China from 2007 to 2011 at the town level [14]. The distribution of the identified cluster was described in the article. Zheng et al. mapped the incidence rate and coefficient of determinants of HFMD to present and identify the most severely affected areas and the most influential determinants [15]. As shown in the study by Wang et al., the typhoid prevalence is spatially clustered and exhibits a gradually decreasing trend [16]. According to Ma et al., bacillary dysentery is not equally distributed across Sichuan Province in China [17]. Nie et al. used a spatial correlation analysis to explore the associations between selected factors and bacillary dysentery in Guangxi Province in China [18]. The spatial analysis visually displayed the discrepancies among different regions, which would be able to present additional results if combined with the temporal analysis.

In conclusion, temporal and spatial analyses are useful. Previous studies have focused on the epidemiological characteristics of different IIDs. However, a comprehensive study analysing the spatial and temporal distributions of all reported IIDs in China is lacking. Second, an overall introduction to IIDs in China to researchers in other countries is unavailable. Therefore, this study will collect data on the basic IIDs and populations, use seasonal decomposition analyses to explore the temporal epidemiology, and perform spatial autocorrelation analysis and space-time scan analysis to explore the spatial epidemiology.

## Methods

### Study setting and data resources

Among the eight IIDs, cholera and poliomyelitis have been almost completely eradicated. The number of deaths related to cholera and poliomyelitis since 2004 were 2180 and 0, respectively, indicating that the two diseases were less emergent. At the same time, according to the Chinese classification of viral hepatitis, the Hepatitis A and E should be analysed separately [19]. Overall, this study analyses the spatiotemporal distribution of six intestinal infectious diseases, namely, dysentery, typhoid and paratyphoid (TAP), viral Hepatitis A, viral Hepatitis E, hand-foot-mouth disease (HFMD) and other infectious intestinal diseases (OIID). To better demonstrate the trends of incidence of IIDs, we analysed these diseases from 2006 to 2016, which represents two equal periods: 2006–2011 and 2011–2016.

The data were collected from public health science data centre issued by Chinese Center for Diseases Control and Prevention (China CDC) [20]. The sorted data are presented in Additional file 2 and Additional file 3. Additional file 2 displays the number of cases of IIDs. Additional file 3 displays the number of cases of IIDs by month and the morbidity of IIDs by month. Intestinal infectious diseases are strictly monitored by the Chinese CDC. The centre aims to integrate the scattered data distributed by governments, universities, research institutes and scientists.

### Temporal analysis method

#### Seasonal decomposition analysis

The seasonal decomposition analysis was based on the seasonal trend decomposition using Loess (STL), which incorporates three components: trend, seasonal, and remainder or residual [21]. Advantages of the method include its simplicity and speed of computation, the robustness of results, and flexibility in the period of the seasonal component [22].

In the present study, IIDs were analysed by performing a seasonal decomposition of the time series. This assay employed Holt-Winters filtering and Ljung-Box test to define the structure (additive or multiplicative) and seasonality (stationary or non-stationary). Using an additive model, the IID results were compiled by summing three components:

$$ {Z}_t={M}_t+{S}_t+{R}_t $$

where *Z*_*t*_ represents the monthly incidence rates of the diseases, *M*_*t*_ represents the trend, *S*_*t*_ symbolizes the seasonal variation, and *R*_*t*_ denotes the remainder.

The result of seasonal decomposition analysis is displayed in a figure with the time from 2006 to 2016 on the horizontal axis and the incidence rate on the vertical axis.

### Spatial analysis method

#### Spatial autocorrelation analysis

The concept of spatial autocorrelation was proposed by Tobler in the first geography law [23]: “Everything is related to everything else, but nearest things are more related than distant things.” Moran’s I was the tool used to measure spatial autocorrelation and consists of two types: global Moran’s I and local Moran’s I. Global Moran’s I measures the general spatial autocorrelation and the spatial distribution of research object. Local Moran’s I reflects the local spatial autocorrelation and the cluster regions. In the present study, spatial autocorrelation was applied to analyse the IIDs. Global Moran’s I shows the overall cluster level and distribution of IIDs; local Moran’s I reveals the specific cluster regions and cluster categories and the hotspots of IIDs [24].

Global Moran’s I is an index ranging from − 1 to 1. When the index was distributed around − 1, the overall spatial distribution displayed the dissimilarity, indicating that high cluster regions bordered on low cluster regions. When the index was approximately 0, a distinct spatial cluster was not observed in the studied regions. When the index was close to 1, the overall spatial distribution revealed the similarity, indicating that the same cluster category was bounded on another cluster category. The following equation was used to calculate the global autocorrelation:

$$ \mathrm{I}=\frac{n{\sum}_{i=1}^n{\sum}_{j=1}^n{w}_{ij}\left({x}_i-\overline{x}\right)\left({x}_j-\overline{x}\right)}{\sum_{i=1}^n{\sum}_{j=1}^n{w}_{ij}{\left({x}_i-\overline{x}\right)}^2} $$

where n denotes the number of observed values, *x*_*i*_ represents the incidence rate in province I, *x*_*j*_ represents the incidence rate in province j, $\overline{x}$ indicates the mean value, and *w*_*ij*_ represents a spatial weight matrix of systematic binomial distribution, which represents neighbouring relations between geographical units with n representing the total number of those units . In the present study, the data were based on regions. The value for *w*_*ij*_ is 1 if province i and province j are adjacent. Otherwise, the value is 0.

Local Moran’s I avoids the weaknesses of global spatial autocorrelation by analysing the spatial autocorrelation of certain characters in local regions. The range and explanation for the local Moran’s I was same as the global index. The cluster results obtained from local Moran’s I were classified into four types: high-high cluster (HH, which indicated that the high cluster areas were surrounded by other high cluster areas), high-low cluster (HL, which indicated that the high cluster areas were surrounded by low cluster areas), low-high cluster (LH), and low-low (LL) cluster. The clusters were visualized using LISA cluster maps. The following equation was used to calculate local Moran’s I:

$$ \mathrm{I}=\frac{n}{S_0}{\sum}_i{\sum}_j{W}_{ij}\left({y}_i-\overline{y}\right)\left({y}_j-\overline{y}\right)/{\sum}_i{\left({y}_i-\overline{y}\right)}^2 $$

$$ {S}_0={\sum}_i{\sum}_j{W}_{ij} $$

where *y*_*i*_ represents the incidence rate in province I, *y*_*j*_ represents the incidence rate in province j, $\overline{y}$ indicates the mean value. [25]

In the present study, we analysed a long period to investigate the incidence rates of different IIDs. The years 2006, 2011, 2016 were selected to show the long-term changes.

#### Space-time scan analysis

Space-time scan statistics were introduced by Kulldorff [26]. The space-time scan statistic based on the discrete Poisson model was applied to detect the space-time cluster of IID cases in high-risk or low-risk regions in China [27]. The shape of space-time scanning windows is cylindrical with the geographic units and the height associated with time [28]. The null hypothesis presumed that the window area and outside areas have the same relative risk (RR) of incidence. The difference in the incidence inside and outside the windows was evaluated by calculating the log likelihood ratio (LLR):

$$ \mathrm{LLR}=\log \left\{{\left(\frac{C}{n}\right)}^c{\left[\frac{\left(C-c\right)}{\left(C-\mathrm{n}\right)}\right]}^{\left(C-c\right)}\right\} $$

where C denotes the total number of cases, c represents the number of observed cases inside the window, and n represents the number of expected cases inside the window [19].

The space-time analysis was applied to identify the clusters according to the LLR value, including the most likely cluster, secondary cluster 1, secondary cluster 2, secondary cluster 3, and secondary cluster 4, as well as the cluster time. Statistical significance was evaluated using a Monte Carlo simulation with 99,999 replicates and a significance level of 0.05. For the other parameters, the maximum radius of the circular base was set to 50% of the total population at risk and the maximum height of the cylinder was set to 50% of the total study period.

The method is sensitive to user-controlled parameter choices. A reliability analysis was conducted to determine the sensitivity and consistency of the results. Multiple scans with different maximum sizes ranging from 50 to 1% of the population were conducted. Reliability was measured using the following equation:

$$ {R}_i={C}_i/S $$

where *R*_*i*_ denotes the reliability of different provincial units, *S* indicates the number of scans, and *C*_*i*_ represents the number of high-risk areas in these scans. The range of reliability is from 0 to 1 points, where 1 indicates that all scans report a place as high risk and 0 indicates no scan reports [29].

The reliability results were visualized by constructing a map (Additional file 4 and Additional file 5).

### Software tools

The seasonal decomposition analysis were visualized using Microsoft Excel (version 2013, Microsoft Corp, Redmond, WA, USA). The seasonal decomposition analysis was performed using IBM SPSS Statistics (version 22, IBM, Armonk, NY, USA). GeoDa (version 1.8.61, GitHub, San Francisco, CA, USA) was employed to attain the global Moran’s I and local Moran’s I hierarchical maps, and LISA cluster maps, and space-time scan maps were obtained using ArcGIS (version 10.0, ESRI Inc., Redlands, CA, USA). The space-time scan was analysed using SatScan (version 9.5, Kulldorff and Information Management Services, Inc., Boston, MA, USA).

## Results

### The prevalence of IIDs

We listed all IIDs reported in each provincial unit in China from 2006 to 2016.

Table 1 presents a descriptive analysis of 6 selected IIDs, including the average, maximum and minimum incidence from 2006 to 2016. All provincial units were divided into east, central and west China.

**Table 1:** The total incidence rates of some IIDs in China from 2006-2016 (1/100000)

Region	HFMD			OIDD			TAP			Dysentery			Hepatitis A			Hepatitis E		
	Incidence	Max	Min	Incidence	Max	Min	Incidence	Max	Min	Incidence	Max	Min	Incidence	Max	Min	Incidence	Max	Min
Beijing	146.32	258.74	14.36	265.38	409.78	181.98	0.09	0.14	0.03	112.85	235.42	41.43	0.94	2.12	0.41	2.26	4.37	1.13
Tianjin	102.92	229.43	0.32	332.58	614.38	214.92	0.16	0.74	0.03	82.55	150.25	51.79	0.38	0.82	0.09	1.59	3.53	0.65
Hebei	82.26	157.59	1.65	72.93	80.64	66.82	0.37	0.46	0.25	23.58	41.83	11.57	1.15	2.29	0.54	1.26	1.64	1.01
Liaoning	57.18	99.95	0.22	37.18	47.59	16.99	0.48	0.75	0.24	17.33	30.61	9.41	3.63	6.76	1.73	3.27	5.21	2.21
Shanghai	158.74	267.84	16.21	24.48	31.89	16.95	0.25	0.63	0.08	6.50	22.63	0.54	1.18	2.39	0.54	2.57	3.11	1.89
Jiangsu	109.57	210.52	0.38	16.90	20.06	11.85	0.42	0.90	0.20	9.71	21.92	4.06	1.57	3.15	0.70	4.29	5.20	3.45
Zhejiang	178.88	386.57	1.62	204.53	264.02	156.45	1.97	5.11	0.70	14.59	41.01	3.95	1.94	4.69	0.84	3.80	4.81	3.16
Fujian	146.78	295.30	0.67	57.63	84.05	38.94	1.25	1.85	0.86	3.74	7.96	1.28	2.25	3.74	1.18	2.32	3.32	1.42
Shandong	86.84	149.38	3.28	43.23	75.42	25.50	0.11	0.38	0.04	10.80	20.02	4.43	0.59	1.14	0.33	1.27	1.66	0.90
Guangdong	217.89	403.50	0.73	114.78	156.95	91.81	1.59	1.91	1.37	6.14	11.27	2.60	1.58	1.96	1.29	2.51	3.03	1.65
Hainan	339.35	760.68	0.06	40.03	54.58	27.02	0.30	0.39	0.21	9.48	16.91	3.43	2.95	8.15	0.69	1.26	4.60	0.21
East China	147.88	–	–	109.97	–	–	0.64	–	–	27.02	–	–	1.65	–	–	2.40	–	–
Shanxi	63.09	97.74	0.49	39.30	53.48	28.94	1.11	2.31	0.69	16.62	29.68	8.95	2.62	3.39	1.77	1.12	1.96	0.33
Jilin	48.72	105.27	0.34	5.83	12.94	2.49	0.06	0.11	0.03	11.79	19.10	3.00	1.37	3.88	0.60	1.08	1.53	0.79
Heilongjiang	30.09	94.74	1.26	13.34	17.64	8.80	0.08	0.12	0.02	14.14	24.49	6.71	1.02	2.21	0.59	1.23	1.47	0.95
Anhui	126.77	248.41	0.26	92.86	149.08	49.19	0.40	0.62	0.26	18.82	25.16	12.41	1.81	3.24	0.82	2.53	3.59	1.25
Jiangxi	66.78	133.70	0.03	61.58	82.25	42.07	0.84	1.60	0.51	17.58	29.24	8.95	2.53	6.82	0.60	1.20	1.82	0.74
Henan	71.86	137.13	0.20	42.75	64.27	28.54	0.21	0.37	0.13	18.27	25.99	12.69	2.78	7.36	0.24	0.58	0.75	0.33
Hubei	101.12	207.14	0.07	44.02	73.03	20.28	0.64	0.99	0.36	17.83	29.28	7.13	2.57	4.55	1.43	3.40	4.68	2.42
Hunan	152.13	318.05	0.09	34.93	45.57	18.38	1.69	2.33	1.31	12.92	17.81	5.65	1.77	4.24	0.84	1.30	2.02	0.46
Central China	82.57	–	–	41.83	–	–	0.63	–	–	16.00	–	–	2.06	–	–	1.56	–	–
Neimenggu	59.43	133.62	0.53	10.78	17.02	7.25	0.13	0.21	0.08	10.71	18.41	5.73	1.77	4.39	0.79	0.53	0.76	0.31
Guangxi	316.89	709.87	0.10	75.05	177.53	44.46	2.46	3.10	2.07	14.72	33.00	6.71	2.51	4.32	1.18	1.08	1.53	0.79
Chongqing	85.44	216.77	0.00	87.01	121.93	56.09	0.49	0.95	0.25	30.90	41.11	23.86	4.89	10.44	2.91	1.77	3.31	0.48
Sichuan	53.73	117.87	0.41	42.60	58.85	30.53	0.41	1.02	0.24	18.13	39.26	6.98	5.15	9.33	2.43	1.03	1.45	0.52
Guizhou	76.87	162.90	0.00	20.76	26.60	14.51	3.77	9.95	1.50	23.64	50.83	8.05	6.64	17.71	0.78	0.94	1.73	0.20
Yunnan	94.75	181.83	0.02	24.81	40.26	13.06	10.45	18.36	4.84	16.01	28.04	8.91	6.78	16.04	2.10	1.61	3.03	0.73
Xizang	27.58	67.20	0.00	2.86	10.83	1.20	0.13	0.85	0.00	43.95	92.79	20.97	8.31	17.99	2.42	0.10	0.24	0.03
Shaanxi	104.77	186.54	0.10	56.09	71.43	42.46	0.09	0.12	0.07	27.19	47.46	11.73	2.05	4.14	0.78	0.62	0.90	0.38
Gansu	32.33	55.81	0.22	36.01	56.96	22.31	0.21	0.31	0.14	42.32	72.11	23.38	9.96	20.71	2.61	0.42	0.64	0.19
Qinghai	26.07	81.82	0.02	50.26	78.35	21.25	0.18	0.34	0.04	20.67	44.18	13.44	11.43	22.17	5.62	1.06	1.99	0.31
Ningxia	77.02	141.88	0.70	128.86	147.03	109.09	0.22	0.34	0.11	49.02	121.04	19.65	9.85	38.92	1.42	0.45	0.77	0.24
Xinjiang	26.95	49.02	1.02	75.65	93.62	62.61	2.23	4.81	0.58	44.62	78.42	16.10	18.69	39.83	8.88	1.25	2.27	0.37
West China	81.82	–	–	50.90	–	–	1.73	–	–	28.49	–	–	7.34	–	–	0.91	–	–
SUM	105.46	–	–	69.52	–	–	1.06	–	–	24.75	–	–	3.96	–	–	1.60	–	–

According to the descriptive summary, HFMD was the most severe disease, with an average incidence of 105.46. OIDD ranked second with an average incidence of 69.52 from 2006 to 2016. The third most common disease was dysentery, with an average incidence of 24.75. Regarding the region, east China had the highest incidence rates for HFMD, OIDD and Hepatitis E. The west area was the area with the greatest number of TAP, dysentery and Hepatitis A cases.

### Seasonal decomposition analysis

The seasonal decomposition plots contain four parts, the raw data, remainder, seasonal variation, and trend. The raw data represent the original incidence rate of certain diseases. The remainder represents the irregularity of data. Seasonal variation exists when the rangeability is greater than 0.2. The trend symbolizes the long-term variation in the data. The seasonality and variation in the trends in monthly data were distinguished from the remainder or residual using a decomposition analysis.

**Fig. 1:**
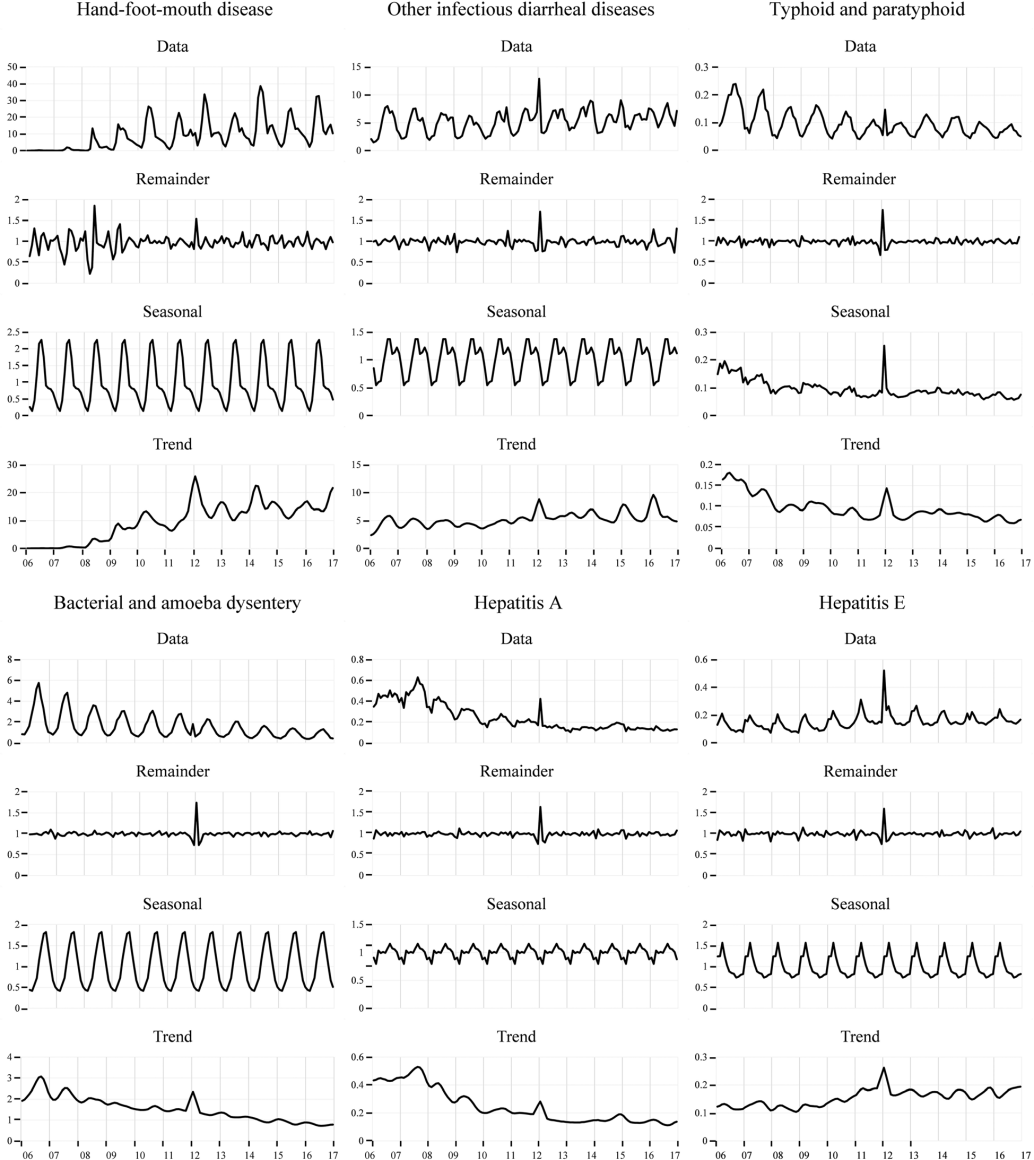
The seasonal decomposition analysis of reportable intestinal infectious diseases

Figure 1 shows the results of the decomposition analysis for all IIDs. Regarding the seasonal variation, the incidence rates of HFMD and dysentery were high in summer. Higher incidence rates for OIDD and Hepatitis A were observed in summer and autumn. Hepatitis E showed a high rate in spring. A distinct seasonal variation in the incidence of TAP was not observed. Regarding the trend, the HFMD, OIDD and Hepatitis E displayed increasing trends, and the other diseases showed decreasing trends. Based on the remainder, a 12-month stochastic variance was displayed.

### Spatial autocorrelation analysis

The spatial autocorrelation analysis was divided into global spatial autocorrelation and local spatial autocorrelation. The former was considered to represent geographical difference in whole areas, while the latter was regarded as the difference at the regional cluster level.

#### Global spatial autocorrelation

Table 2 shows the results from the global spatial autocorrelation analysis and significance test. In terms of the significance, all Moran’s I values for dysentery and Hepatitis E reached the significance threshold, while of the values for OIDD, TAP, and Hepatitis A achieved results, with one insignificant index each. HFMD had five significant indexes. In the global spatial autocorrelation analysis, Moran’s I for HFMD ranged from 0.0039 to 0.3812 throughout the 11 years without an obvious increasing or decreasing trend, suggesting that the disease did not show a difference over time. Moran’s I for OIDD ranged from 0.1054 to 0.3828, showing a descending trend over the years and exhibiting a weakening geographical difference. TAP had a Moran’s I ranging from 0.0879 to 0.2583 with an increasing trend, which implicated a stronger spatial autocorrelation and geographical difference over time. Dysentery had a Moran’s I ranging from 0.3926 to 0.4704, with a slight fluctuation indicating the persistently high difference among provinces. The morbidity rates of Hepatitis A and E exhibited decreasing trends in the geographical difference, with ranges of 0.2434–0.6439 and 0.0735–0.3468, respectively.

**Table 2:** Global spatial autocorrelation analysis and significance rest results

Year	Hand-foot-mouth diseases		Other infectious diarrhoeal diseases		Typhoid and paratyphoid		Bacterial and amoebic dysentery		Hepatitis A		Hepatitis E	
	Moran’s I	*p*-value	Moran’s I	p-value	Moran’s I	*p*-value	Moran’s I	p-value	Moran’s I	p-value	Moran’s I	*p*-value
2006	0.0039	0.1996	0.3828***	0.0033	0.1758**	0.0254	0.4082***	0.0010	0.5233***	0.0001	0.3468***	0.0030
2007	0.0320	0.2049	0.3718***	0.0045	0.1514**	0.0397	0.3926***	0.0010	0.3893***	0.0021	0.3155***	0.0042
2008	0.2057**^1^	0.0257	0.3083***	0.0077	0.2281**	0.0107	0.4133***	0.0013	0.5435***	0.0001	0.2948***	0.0057
2009	0.3812***	0.0004	0.3710***	0.0039	0.1558**	0.0249	0.4189***	0.0005	0.4891***	0.0002	0.3033***	0.0050
2010	0.0844	0.1552	0.2884***	0.0099	0.1198**	0.0332	0.4138***	0.0004	0.4441***	0.0003	0.2871***	0.0063
2011	0.0587	0.1644	0.2126**	0.0259	0.1618	0.1236	0.4477***	0.0003	0.5390***	0.0004	0.2783***	0.0076
2012	0.1664**	0.0441	0.2313**	0.0178	0.1461**	0.0124	0.4588***	0.0003	0.6439***	0.0001	0.1201*	0.0719
2013	0.0776	0.1373	0.1781**	0.0396	0.1350**	0.0175	0.4619***	0.0004	0.6047***	0.0002	0.1761**	0.0406
2014	0.2061**	0.0212	0.2344**	0.0169	0.0879**	0.0343	0.4704***	0.0004	0.2434***	0.0008	0.1459*	0.0673
2015	0.0598	0.1539	0.1814**	0.0375	0.1876***	0.0100	0.4545***	0.0005	0.3931***	0.0006	0.1991**	0.0295
2016	0.2288**	0.0159	0.1054	0.1181	0.2583***	0.0074	0.4444***	0.0006	0.1924**	0.0164	0.0735	0.1784

^1^ *a 10% level of statistical significance; **a 5% level of statistical significance; ***a 1% level of statistical significance

#### Local spatial autocorrelation analysis

**Fig. 2:**
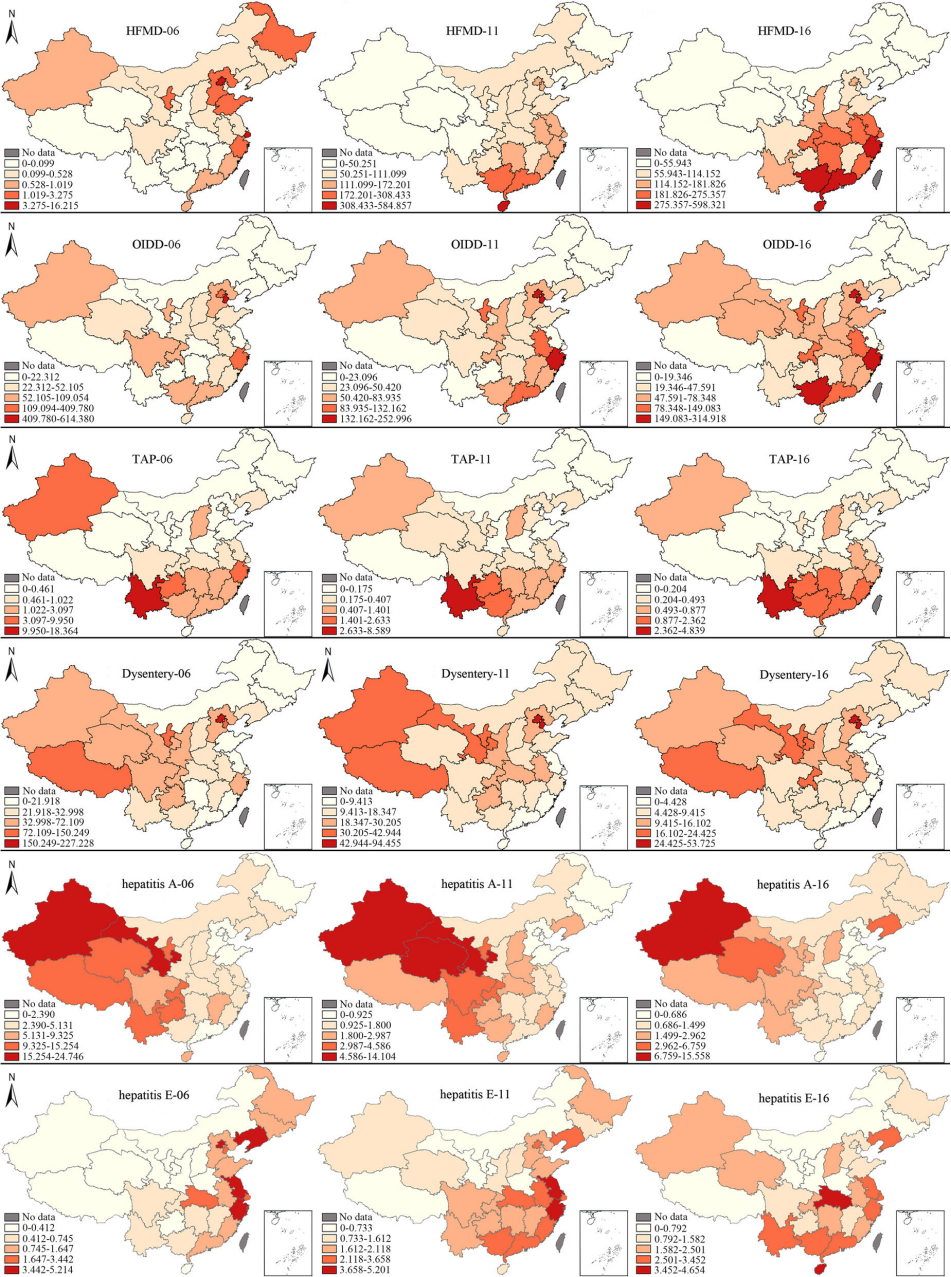
Map showing the hierarchy of the incidence rates for IIDs in 2006, 2011 and 2016

Figure 2 shows the incidence rates of all IIDs in different provinces from 2006 to 2016, as represented by the data from 2006, 2011 and 2016. The map showing the hierarchy of the incidence rate was classified into five layers according to the severity of diseases, which were based on the maximum value for a specific disease. A deeper red colour indicates a higher incidence rate. Using the incidence of HFMD in 2006 as an example, the maximum incidence of HFMD in 2006 was 16.215, and the five classes ranged from 0 to 16.215. The range of the classes were 0–0.099, 0.099–0.528, 0.528–1.019, 1.019–3.275, and 3.275–16.215. Beijing and Shanghai were the areas with the highest incidence rates, while the areas with the lowest rates included Xizang, Qinghai, Yunnan, and Guangxi, among others.

**Fig. 3:**
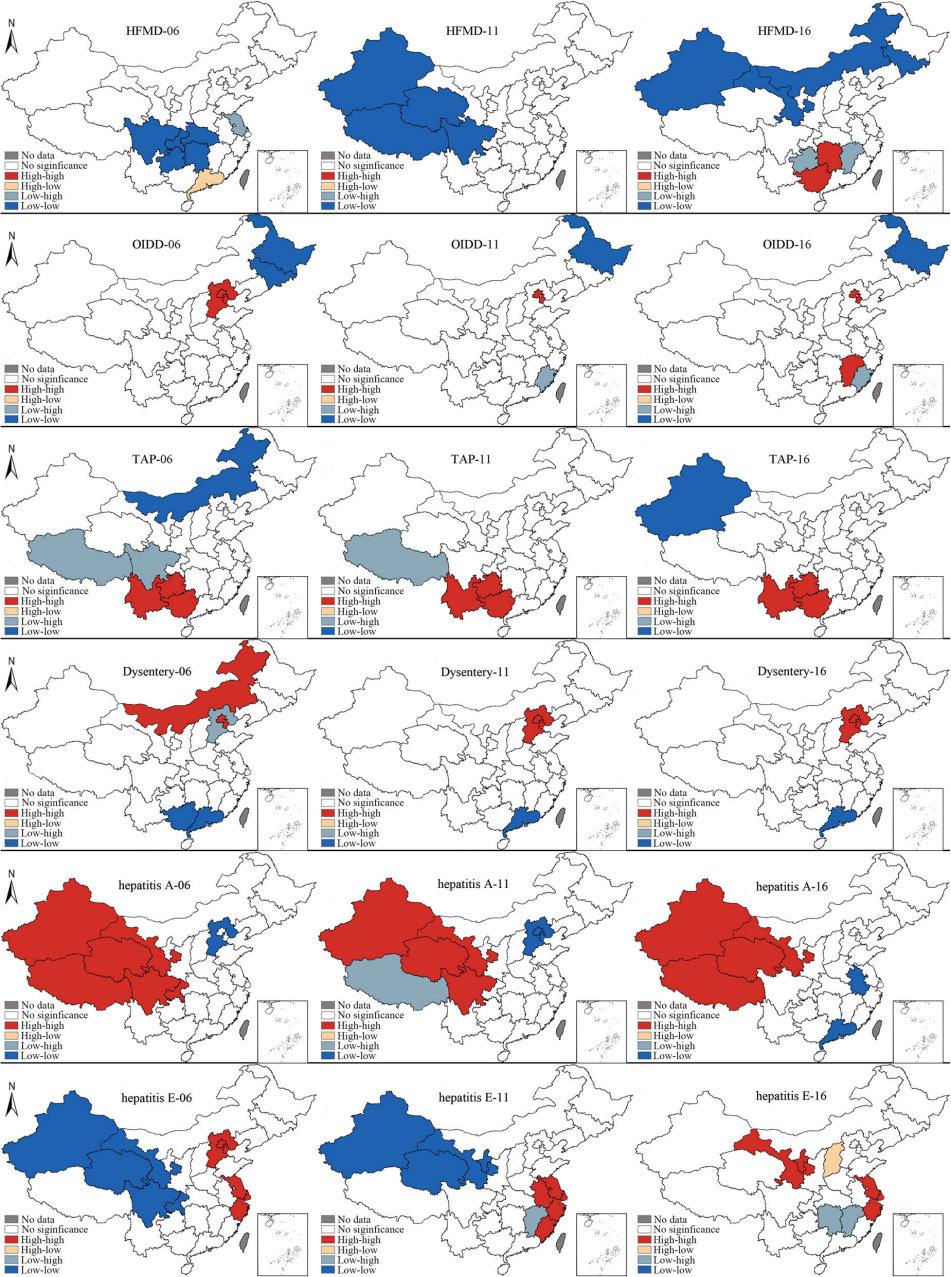
The spatial clusters of IIDs in 2006, 2011 and 2016

Figure 3 displays the spatial clusters of all IIDs, reflecting the regional variation from 2006 to 2016. The hot spot for HFMD is located in Guangdong, while cold spots were detected in some areas of the central and west provinces, such as Sichuan, Chongqing, Hubei, Guizhou, and Jiangxi. In 2016, the hot spots shifted to adjacent provinces, Guizhou and Jiangxi; new cold spots shifted to areas in the north province, such as Gansu, Jilin, Xinjiang, and Inner Mongolia.

For OIDD, the HH cluster feature was observed in BTT areas, while Heilongjiang and Jilin in northwest China showed the LL cluster feature in 2006. Throughout the ensuing 10 years, Jilin and Hebei were no longer present in the original hot spots and cold spots. Jiangxi showed the HH cluster character as well.

For TAP, HH cluster character was witnessed in Yunnan, Jiangxi, and Guizhou throughout the 10-year period, while Xizang, Sichuan, Neimenggu and Xinjiang showed the character of a cold spot.

In terms of dysentery, Inner Mongolia, Beijing, and Tianjin were located in HH cluster areas, while the LH cluster was located in Hebei. Guangxi and Guangdong contained the LL cluster. In 2011 and 2016, the cluster characters presented the same trends. Beijing, Tianjin, and Hebei were the HH cluster areas, while the LL cluster was located in Guangdong. Hebei experienced a change from a cold spot to a hot spot.

For Hepatitis A, the HH cluster character was present in west China, including Xinjiang, Gansu, Qinghai, Xizang, Sichuan, and Chongqing, throughout the 10-year period, while the LL cluster character was located in the BTT area, Jiangsu and Guangdong. Xizang was a hot spot in 2006 and 2016, but was a cold spot in 2011.

The hot spots for Hepatitis E were mainly concentrated in the BTT area and Yangtze River Delta area, while cold spots were located in areas of west China, Jiangxi and Hubei.

### Space-time scan analysis

**Fig. 4:**
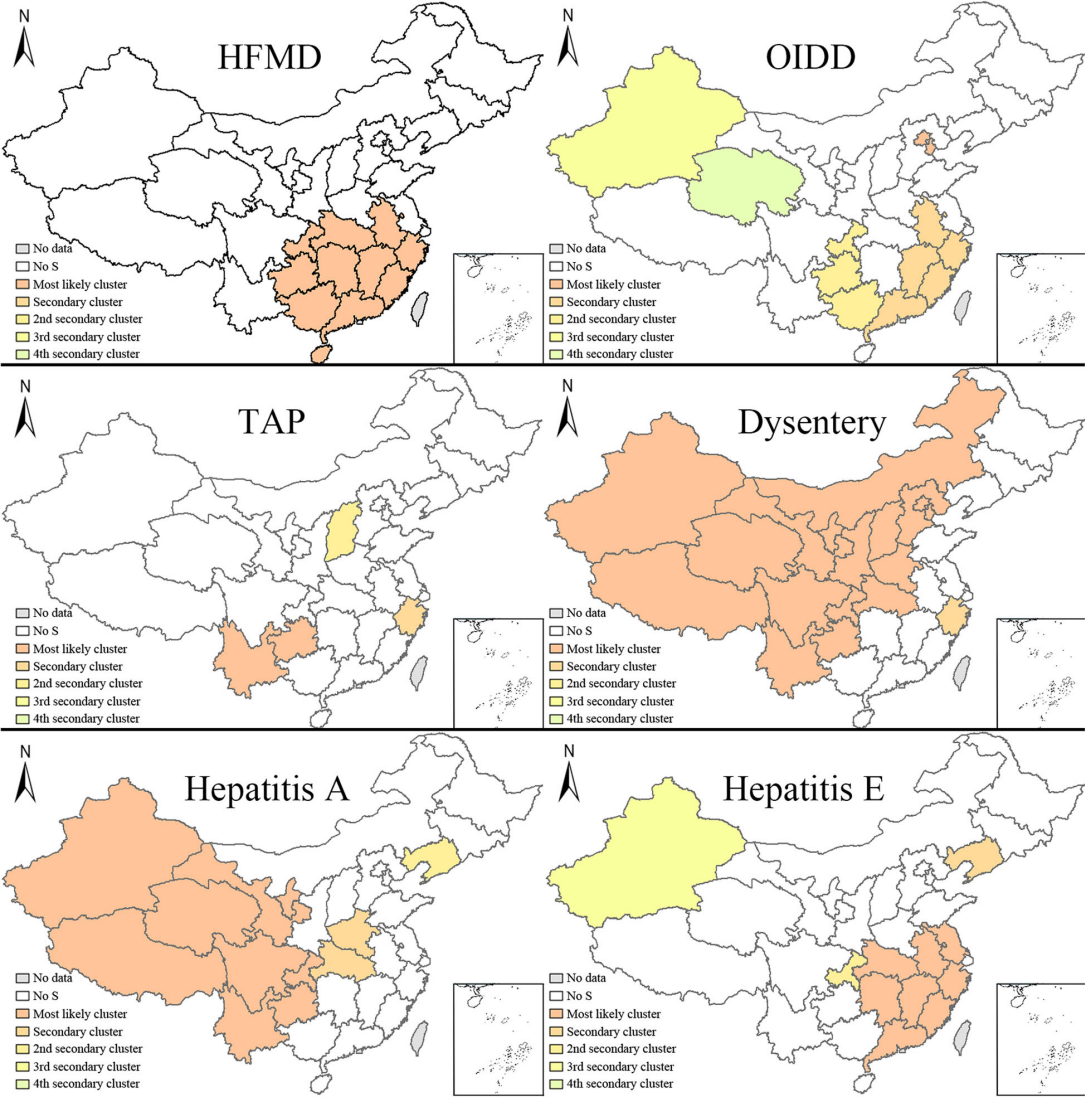
Space-time analysis of IIDs with 50% of the population at risk. Note:S indicates significance

Space-time scan analysis was used to explore the cluster likelihood, the level of which was classified as the most likely cluster, secondary cluster, 2nd secondary cluster, 3rd secondary cluster and 4th secondary cluster. This study analysed IIDs in China from 2006 to 2016. Fig. 4 and  Table 3 displaysed the space-time scan analysis of all IIDs in 2016 with 50% of the population at risk.

**Table 3:** Space-time analysis of IIDs with 50% of the population at risk

Type of diseases	Cluster type	Location center	Cluster areas	Coordinates	Radius(Km)	Time(Year)	Number of Cases	Expected Cases	Annual Cases/10000	Relative Risk	LLR	*p*-Value
HFMD	Most likely cluster	Guangdong	11	23.28 N, 113.36E	1041.22	2012–2016	7,127,929	3,082,303.41	257.0	3.33	2,621,208.06	<0.001
OIDD	Most likely cluster	Beijing	2	40.22 N, 116.44E	135.55	2006–2010	499,320	90,604.72	343.6	5.77	452,831.47	<0.001
OIDD	Secondary cluster	Fujian	5	26.00 N, 118.02E	672.79	2012–2016	1,845,235	958,099.52	120.1	2.16	371,972.73	<0.001
OIDD	2nd secondary cluster	Guizhou	3	26.67 N, 106.61E	444.67	2015–2016	224,173	142,078.48	98.4	1.59	20,511.25	<0.001
OIDD	3rd secondary cluster	Xinjiang	1	42.00 N, 85.66E	0	2006–2009	73,852	52,610.52	87.5	1.41	3829.96	<0.001
OIDD	4th secondary cluster	Qinghai	1	35.72 N, 96.48E	0	2012–2016	20,305	18,177.75	69.6	1.12	120.13	<0.001
TAP	Most likely cluster	Yunnan	2	24.14 N, 101.30E	602.35	2006–2010	39,400	4665.15	9.6	10.74	53,337.37	<0.001
TAP	Secondary cluster	Zhejiang	1	29.10 N, 120.10E	0	2006–2008	6297	1726.13	4.1	3.75	3642.35	<0.001
TAP	2nd secondary cluster	Shanxi	1	37.70 N, 112.38E	0	2014	837	414.40	2.3	2.02	166.34	<0.001
dysentery	Most likely cluster	Xinjiang	17	42.00 N, 85.66E	2723.61	2006–2010	1,053,358	545,351.07	35.1	2.54	251,530.50	<0.001
dysentery	Secondary cluster	Zhejiang	1	29.10 N, 120.10E	0	2006–2008	48,455	27,597.65	31.9	1.77	6500.61	<0.001
Hepatitis A	Most likely cluster	Xinjiang	9	31.10 N, 89.12E	1790.28	2006–2010	142,703	36,975.74	11.3	5.28	102,737.42	<0.001
Hepatitis A	Secondary cluster	Henan	2	33.80 N, 113.59E	326.52	2006–2009	29,048	17,636.02	4.8	1.69	3242.92	<0.001
Hepatitis A	2nd secondary cluster	Liaoning	1	41.47 N, 123.52E	0	2015–2016	4919	2556.40	5.6	1.93	863.46	<0.001
Hepatitis E	Most likely cluster	Jiangxi	8	27.73 N, 115.63E	671.06	2010–2014	76,951	46,212.61	3.0	1.93	10,730.60	<0.001
Hepatitis E	Secondary cluster	Liaoning	1	41.47 N, 123.52E	0	2006–2010	8095	3941.52	3.7	2.09	1705.11	<0.001
Hepatitis E	2nd secondary cluster	Chongqing	1	29.80 N, 107.77E	0	2013–2016	3485	2194.17	2.9	1.60	324.69	<0.001
Hepatitis E	3rd secondary cluster	Xinjiang	1	42.00 N, 85.66E	0	2015	522	429.88	2.2	1.21	9.25	<0.001

The most likely cluster areas for HFMD were located in south China (Guangdong, Guangxi, Jiangxi, Hunan, Fujian, Hainan, Guizhou, Hubei, Chongqing, Zhejiang, and Anhui) from 2012 to 2016, and no other levels were detected.

The most likely cluster areas for OIDDs were Beijing and Tianjin from 2006 to 2010; secondary clusters were located in southwest China (Fujian, Jiangxi, Zhejiang, Guangdong, and Anhui) from 2012 to 2016 and were located in some areas of southwest China (Guizhou, Chongqing, and Guangxi) from 2015 to 2016. Third secondary clusters were observed in Xinjiang Province from 2006 to 2009. The 4th secondary cluster was located in Qinghai from 2012 to 2016.

The most likely cluster areas for TAP were detected Yunnan and Guizhou located in southwest China from 2006 to 2010. The secondary cluster areas were located in Zhejiang Province from 2006 to 2008. Shanxi Province was the location of the 2nd secondary cluster in 2014.

For dysentery, the most likely cluster was located in central and west China (Xinjiang, Qinghai, Xizang, Gansu, Ningxia, Sichuan, Neimenggu, Shaanxi, Shanxi, Chongqing, Yunnan, Hebei, Guizhou, Beijing, Henan, Tianjin, and Hubei) from 2006 to 2010. Zhejiang Province was the site of the secondary cluster from 2006 to 2008.

For Hepatitis A, west China (Xizang, Qinghai, Xinjiang, Sichuan, Yunnan, Gansu, Ningxia, Guizhou, and Chongqing) was the first cluster area from 2006 to 2010. Henan and Hubei had the character of a secondary cluster from 2006 to 2009. Liaoning had the character of the 1st secondary cluster.

The first cluster for Hepatitis E was located in south China (Jiangxi, Fujian, Hunan, Hubei, Zhejiang, Anhui, Guangdong, and Jiangsu) from 2010 to 2014. Liaoning had the character of a secondary cluster from 2006 to 2010. Chongqing had the character of a 1st secondary cluster from 2013 to 2016. Xinjiang was considered a 2nd secondary cluster in 2015.

## Discussion

Studies of the epidemiology of different intestinal infectious diseases, particularly the temporal and spatial distributions, have played an important role in preventing infections prevention. However, previous studies have neglected to analyse the correlation between the temporal and spatial analyses. A systematic spatio-temporal analysis of IIDs has not been conducted in China. This study supplements data from other related research in the area of infectious intestinal diseases by performing a temporal analysis and spatial analysis and determining the correlation between them. The evidence provided insights into potential solutions to diminish the diseases.

On one hand, we would like to compare the temporal analyses of the six intestinal infectious diseases. According to the incidence rates recorded from 2006 to 2016, the temporal trends differed. Regarding the absolute incidence of cases, the incidence of HFMD was higher than the other IIDs, and it gradually became a wide-spread disease, which is consistent with the results from the study by Zhang [30]. Then, dysentery and OIDD were less severe diseases. Hepatitis A, Hepatitis E and TAP were the least severe diseases, according to the incidence rates. Regarding the trend, the incidence rates of HFMD and Hepatitis E showed a distinct increasing trend, consistent with the results from the study by Zhu. As the epidemic of Hepatitis A was controlled, the percentage of Hepatitis E cases among patients with viral hepatitis and among patients with IIDs has increased. The mortality rate of Hepatitis E has increased among infectious diseases [31]. The trend in the incidence of OIDD was almost unchangeable. Dysentery and TAP displayed obvious decreasing trends. The analysis of dysentery filled the research gap in the study by Xie et al., which showed a decreasing trend in the incidence of dysentery from 2005 to 2010 [32]. The results for TAP were similar to the findings reported by Liu [33], which showed a decreasing trend. The improvement in sanitation facilities and the reduction in food and water pollution likely contributed to the decreasing trend in the incidence rates of these diseases. Hepatitis A exhibited a slight increasing trend in the first 2 years, followed by a decreasing trend. This result was similar to the findings reported by Zhu [19]. In conclusion, class B diseases were prevented with high efficiency. Class C diseases experienced a higher incidence rate and gradually increasing trend. Regarding the seasonal changes, the incidence of HFMD and dysentery peaked in the summer, Hepatitis E exhibited a high-incidence season in spring, OIDD peaked in summer and a smaller peak in autumn, the peak incidence of Hepatitis A occurred in summer and spring, and a distinct peak for TAP was not observed. Previous studies have revealed an association between the IIDs and seasons [16, 34–37]. The occurrence of intestinal infectious diseases is related to climatic factors, such as the sunshine duration, temperature and humidity, as well as the quality of food and drinking water [38]. Due to the high temperature and humidity in summer, which are conducive to bacterial reproduction, food and water are easily contaminated. At the same time, the human immune system is relatively weak due to higher bodily exertion in summer. In conclusion, summer is the season with the highest incidence rates for IIDs and should receive closer attention.

On the other hand, the high-risk areas for different IIDs were mainly located in the Beijing-Tianjin-Tangshan (BTT) region, Yangtze River Delta, south and west China. The former two sites are developed areas with extensive urbanization, which attract larger mobile populations characterized by low immune systems, poor living environments and living conditions, and poor health and knowledge of epidemic prevention measures. The hot spots for HFMD, OIDD, dysentery and Hepatitis E are located in the BTT region and Yangtze River Delta [39]. South China is located closer to the equator with a subtropical monsoon and tropical monsoon climate, which are characterized by high humidity, temperature, rainfall, and wind speed. IIDs are strongly correlated with the climatic character of south China [40], which is a high-risk area for HFMD, OIDD, and TAP. The high-risk areas for TAP were located in southwest China (Yunnan, Guizhou, and Guangxi), which are also the most likely cluster areas and HH cluster areas. The main reasons are that southwest China borders Southeast Asia (Vietnam, Laos, and Myanmar), which has a high-risk population due to low sanitation. Moreover, the population of southwest China has a medical history of TAP and very poor climatic, geographical (Karst landform) and sanitation conditions. Finally, personal eating habits contribute to the incidence of TAP [41]. The west area experiences poor sanitation and a lower economy and is a high-incidence area for Hepatitis A and dysentery.

The strengths of this study are listed below. First, the study performed a comprehensive analysis of IIDs, including the temporal and spatial analyses of all reported intestinal diseases in China, to provide a complete picture of IIDs to other countries. Second, the visualization of diseases represented a convenient method to show the distribution of diseases. However, this study had some limitations. We were unable to obtain more sophisticated results for the provincial units in China than were obtained for the county unit. Regional discrepancies were also observed among provinces. Furthermore, additional studies that explore the temporal and spatial distributions in smaller region units are needed.

## Conclusions

In conclusion, seasonal patterns and trends in different provincial geographical units were determined. Higher incidence rates of IIDs were observed from May to October, which is the season with a climate characterized by heavy rain, high temperature, and high humidity. The climate zone is associated with the incidence. The high-risk areas for IIDs were detected in part of the border region, the south region and the region with better economic development.

Based on the results of our temporal and spatial analysis of IIDs, we identified the high-risk periods and cluster regions for the studied diseases. HFMD and OIDD exhibited high incidence rates, reflecting the negligence of the government in monitoring Class C diseases. At the same time, the incidence rate of Hepatitis E gradually surpassed that of Hepatitis A. The authorities should closely monitor Class C diseases and Hepatitis E. Notable epidemiological trends were observed among different provinces. An effective response requires the implementation of a series of coherent and coordinated measures, which should be specific for the diseases that are endemic to a particular area.

## Additional files

Additional file 1: The classification of reported infectious diseases in China. (DOCX 3959 kb)

Additional file 2: The number of cases of IIDs. (XLS 68 kb)

Additional file 3: The number of cases of IIDs by month; The Morbidity of IIDs by month. (XLSX 26 kb)

Additional file 4: The Results of reliability. (DOCX 13 kb)

Additional file 5: The analysis process of reliability. (XLSX 45 kb)

## Data Availability

All data generated or analyzed during this study are included in this published article and its supplementary information files.

## Abbreviations

AHC: Acute hemorrhagic conjunctivitis
AIDS: Acquired immunodeficiency syndrome
BBT: Beijing-Tianjin-Tangshan
CA: California
CDC: Center for Diseases Control and Prevention
EHF: Epidemic hemorrhagic fever
ESRI: Environmental Systems Research Institute
GPEI: Global polio eradication initiative
HFMD: Hand, foot and mouth disease
HH: High-high
HL: High-low
IBM: International Business Machines Corporation
IIDs: Intestinal infectious diseases
LH: Low-high
LISA: Local indicators of spatial association
LL: Low-low
LLR: Log Likelihood Ratio
MA: Massachusetts
NHANES: National Health and Nutrition Examination Survey
NY: New York
OIDD: Other infectious diarrheal diseases
OPV: Oral polio vaccine
R&D: Research and development
RR: Relative risk
SARS: Severe Acute Respiratory Syndrome
STL: Seasonal Trend Decomposition using Loess
TAP: Typhoid and paratyphoid
USA: The United States
WA: Washington
