# Supplementary material for: A descriptive analysis of the Spatio-temporal distribution of intestinal infectious diseases in China
Source: BMC Infect Dis. 2019 Sep 2;19:766. doi: 10.1186/s12879-019-4400-x (PMC6721277; doi:10.1186/s12879-019-4400-x)
Supplement: Supplementary file 1 — The classification of reported infectious diseases in China. (DOCX 3959 kb) [file 12879_2019_4400_MOESM1_ESM.docx]

Additional file 1 the classification of reported infectious diseases in China

| **Classification** | **Included infectious diseases** |
| --- | --- |
| **Class A** | Plague, Cholera; |
| **Class B** | Severe acute respiratory syndrome (SARS), Acquired immunodeficiency syndrome (AIDS), Viral hepatitis, Poliomyelitis, Human infections of highly pathogenic avian influenza, human infections of H7N9 avian influenza, Measles, Epidemic hemorrhagic fever (EHF), Rabies, Epidemic encephalitis B, Dengue, Anthrax, Bacterial and amoebic dysentery, Tuberculosis, Typhoid & paratyphoid , Epidemic (meningococcal) meningitis, Pertussis, Diphtheria, Neonatal tetanus, Scarlet fever, Brucellosis, Gonorrhea, Syphilis, Leptospirosis, Schistosomiasis, Malaria; |
| **Class C** | Influenza, Mumps, Rubella, Acute hemorrhagic conjunctivitis (AHC), Leprosy, Typhus, Leishmaniosis, Echinococcosis, Filariasis, Other infectious diarrheal diseases, Hand, foot and mouth disease (HFMD). |
