# Supplementary material for: A descriptive analysis of the Spatio-temporal distribution of intestinal infectious diseases in China
Source: BMC Infect Dis. 2019 Sep 2;19:766. doi: 10.1186/s12879-019-4400-x (PMC6721277; doi:10.1186/s12879-019-4400-x)
Supplement: Supplementary file 4 — The Results of reliability. (DOCX 13 kb) [file 12879_2019_4400_MOESM4_ESM.docx]

The Results of reliability

Table 1 Space-time analysis of IIDs with 50% of the population at risk

| Type of diseases | Cluster type | Location center | Cluster areas | Coordinates | Radius(Km) | Time(Year) | Number of Cases | Expected Cases | Annual Cases/10000 | Relative Risk | LLR | p-Value |
| --- | --- | --- | --- | --- | --- | --- | --- | --- | --- | --- | --- | --- |
| HFMD | Most likely cluster | Guangdong | 11 | 23.28N, 113.36E | 1041.22 | 2012-2016 | 7127929 | 3082303.41 | 257.0 | 3.33 | 2621208.06 | ＜0.001 |
| OIDD | Most likely cluster | Beijing | 2 | 40.22N, 116.44E | 135.55 | 2006-2010 | 499320 | 90604.72 | 343.6 | 5.77 | 452831.47 | ＜0.001 |
| OIDD | Secondary cluster | Fujian | 5 | 26.00N, 118.02E | 672.79 | 2012-2016 | 1845235 | 958099.52 | 120.1 | 2.16 | 371972.73 | ＜0.001 |
| OIDD | 2^nd^ secondary cluster | Guizhou | 3 | 26.67N, 106.61E | 444.67 | 2015-2016 | 224173 | 142078.48 | 98.4 | 1.59 | 20511.25 | ＜0.001 |
| OIDD | 3^rd^ secondary cluster | Xinjiang | 1 | 42.00N, 85.66E | 0 | 2006-2009 | 73852 | 52610.52 | 87.5 | 1.41 | 3829.96 | ＜0.001 |
| OIDD | 4^th^ secondary cluster | Qinghai | 1 | 35.72N, 96.48E | 0 | 2012-2016 | 20305 | 18177.75 | 69.6 | 1.12 | 120.13 | ＜0.001 |
| TAP | Most likely cluster | Yunnan | 2 | 24.14N, 101.30E | 602.35 | 2006-2010 | 39400 | 4665.15 | 9.6 | 10.74 | 53337.37 | ＜0.001 |
| TAP | Secondary cluster | Zhejiang | 1 | 29.10N, 120.10E | 0 | 2006-2008 | 6297 | 1726.13 | 4.1 | 3.75 | 3642.35 | ＜0.001 |
| TAP | 2^nd^ secondary cluster | Shanxi | 1 | 37.70N, 112.38E | 0 | 2014 | 837 | 414.40 | 2.3 | 2.02 | 166.34 | ＜0.001 |
| dysentery | Most likely cluster | Xinjiang | 17 | 42.00N, 85.66E | 2723.61 | 2006-2010 | 1053358 | 545351.07 | 35.1 | 2.54 | 251530.50 | ＜0.001 |
| dysentery | Secondary cluster | Zhejiang | 1 | 29.10N, 120.10E | 0 | 2006-2008 | 48455 | 27597.65 | 31.9 | 1.77 | 6500.61 | ＜0.001 |
| Hepatitis A | Most likely cluster | Xinjiang | 9 | 31.10N, 89.12E | 1790.28 | 2006-2010 | 142703 | 36975.74 | 11.3 | 5.28 | 102737.42 | ＜0.001 |
| Hepatitis A | Secondary cluster | Henan | 2 | 33.80N, 113.59E | 326.52 | 2006-2009 | 29048 | 17636.02 | 4.8 | 1.69 | 3242.92 | ＜0.001 |
| Hepatitis A | 2^nd^ secondary cluster | Liaoning | 1 | 41.47N, 123.52E | 0 | 2015-2016 | 4919 | 2556.40 | 5.6 | 1.93 | 863.46 | ＜0.001 |
| Hepatitis E | Most likely cluster | Jiangxi | 8 | 27.73N, 115.63E | 671.06 | 2010-2014 | 76951 | 46212.61 | 3.0 | 1.93 | 10730.60 | ＜0.001 |
| Hepatitis E | Secondary cluster | Liaoning | 1 | 41.47N, 123.52E | 0 | 2006-2010 | 8095 | 3941.52 | 3.7 | 2.09 | 1705.11 | ＜0.001 |
| Hepatitis E | 2^nd^ secondary cluster | Chongqing | 1 | 29.80N, 107.77E | 0 | 2013-2016 | 3485 | 2194.17 | 2.9 | 1.60 | 324.69 | ＜0.001 |
| Hepatitis E | 3^rd^ secondary cluster | Xinjiang | 1 | 42.00N, 85.66E | 0 | 2015 | 522 | 429.88 | 2.2 | 1.21 | 9.25 | ＜0.001 |


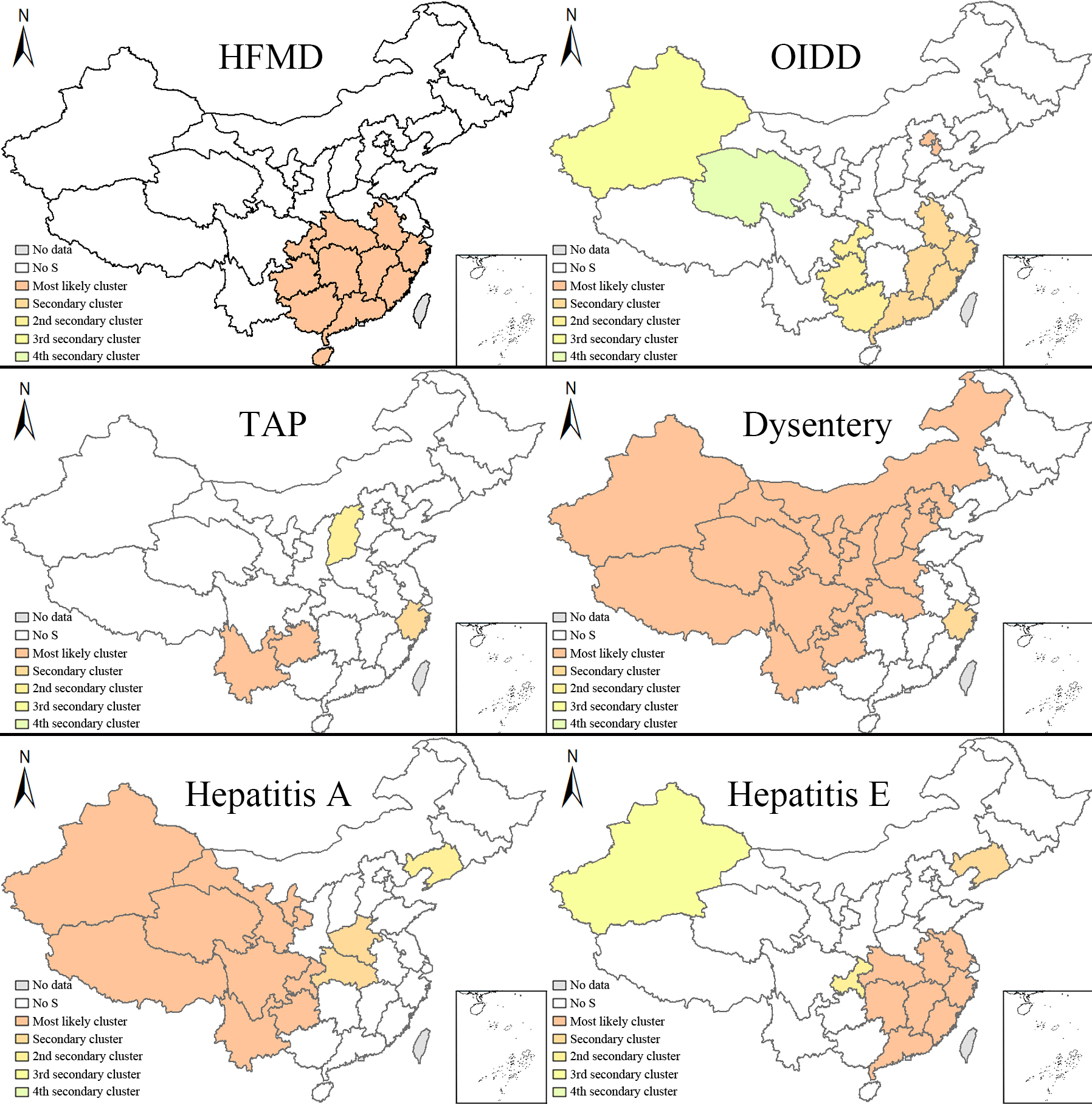


Table 2 Space-time analysis of HFMD

| Maximum Spatial Cluster Size | Cluster type | Location center | Cluster areas | Coordinates | Radius(Km) | Time(Year) | Number of Cases | Expected Cases | Annual Cases/10000 | Relative Risk | LLR | p-Value |
| --- | --- | --- | --- | --- | --- | --- | --- | --- | --- | --- | --- | --- |
| 50% | Most likely cluster | Guangdong | 11 | 23.28N, 113.36E | 1041.22 | 2012-2016 | 7127929 | 3082303.41 | 257.0 | 3.33 | 2621208.06 | ＜0.001 |
| 40% | Most likely cluster | Guangdong | 10 | 23.28N, 113.36E | 933.30 | 2012-2016 | 6539278 | 2743898.85 | 264.8 | 3.31 | 2470430.24 | ＜0.001 |
| 40% | Secondary cluster | Shandong | 9 | 36.18N, 118.43E | 628.08 | 2014 | 816932 | 559601.92 | 162.2 | 1.48 | 53848.07 | ＜0.001 |
| 30% | Most likely cluster | Guangdong | 6 | 23.28N, 113.36E | 583.68 | 2012-2016 | 4883071 | 1751285.00 | 309.8 | 3.55 | 2238307.79 | ＜0.001 |
| 30% | Secondary cluster | Jiangsu | 4 | 32.47N, 119.97E | 374.35 | 2012-2016 | 2229992 | 1221727.01 | 202.8 | 1.96 | 367962.39 | ＜0.001 |
| 30% | 2^nd^ secondary cluster | Beijing | 2 | 40.22N, 116.44E | 135.55 | 2010-2014 | 300709 | 192733.02 | 173.4 | 1.57 | 26152.11 | ＜0.001 |
| 30% | 3^rd^ secondary cluster | Shaanxi | 4 | 34.12N, 108.76E | 488.76 | 2014-2016 | 718592 | 565583.38 | 141.2 | 1.28 | 19792.14 | ＜0.001 |
| 20% | Most likely cluster | Guangxi | 5 | 23.02N, 108.41E | 640.03 | 2012-2016 | 4417238 | 1482412.44 | 331.1 | 3.71 | 2198940.43 | ＜0.001 |
| 20% | Secondary cluster | Zhejiang | 5 | 29.10N, 120.10E | 426.92 | 2012-2016 | 2683837 | 1433379.25 | 208.1 | 2.04 | 486816.13 | ＜0.001 |
| 20% | 2^nd^ secondary cluster | Beijing | 2 | 40.22N, 116.44E | 135.55 | 2010-2014 | 300709 | 192733.02 | 173.4 | 1.57 | 26152.11 | ＜0.001 |
| 20% | 3^rd^ secondary cluster | Shaanxi | 4 | 34.12N, 108.76E | 488.76 | 2014-2016 | 718592 | 565583.38 | 141.2 | 1.28 | 19792.14 | ＜0.001 |
| 20% | 4^th^ secondary cluster | Shanxi | 3 | 37.70N, 112.38E | 424.93 | 2009 | 175728 | 143776.22 | 135.8 | 1.22 | 3344.71 | ＜0.001 |
| 10% | Most likely cluster | Guangxi | 2 | 23.02N, 108.41E | 444.53 | 2012-2016 | 1500122 | 314586.31 | 529.9 | 4.77 | 1202662.50 | ＜0.001 |
| 10% | Secondary cluster | Guangdong | 1 | 23.28N, 113.36E | 0 | 2012-2016 | 1860298 | 598120.22 | 345.6 | 3.38 | 900674.11 | ＜0.001 |
| 10% | 2^nd^ secondary cluster | Zhejiang | 2 | 29.10 N, 120.10E | 279.11 | 2012-2016 | 1009470 | 440944.34 | 254.4 | 2.37 | 277860.81 | ＜0.001 |
| 10% | 3^rd^ secondary cluster | Hunan | 1 | 28.02N, 111.58E | 0 | 2012-2016 | 831644 | 374291.63 | 246.9 | 2.29 | 213215.96 | ＜0.001 |
| 10% | 4^th^ secondary cluster | Fujian | 1 | 26.00N, 118.02E | 0 | 2012-2016 | 453845 | 211652.25 | 238.3 | 2.18 | 105831.61 | ＜0.001 |
| 10% | 5^th^ secondary cluster | Anhui | 1 | 32.01 N, 117.19E | 0 | 2012-2016 | 588651 | 338404.56 | 193.3 | 1.77 | 77591.341160 | ＜0.001 |
| 10% | 6^th^ secondary cluster | Beijing | 2 | 40.22N, 116.44E | 135.55 | 2010-2014 | 300709 | 192733.02 | 173.4 | 1.57 | 26152.11 | ＜0.001 |
| 10% | 7^th^ secondary cluster | Hubei | 1 | 30.90N, 113.03E | 0 | 2012-2016 | 453295 | 323835.22 | 155.6 | 1.41 | 23511.36 | ＜0.001 |
| 10% | 8^th^ secondary cluster | Yunnan | 1 | 24.14N, 101.30E | 0 | 2014-2016 | 251000 | 158103.81 | 176.4 | 1.60 | 23383.26 | ＜0.001 |
| 10% | 9^th^ secondary cluster | Shaanxi | 1 | 34.12N, 108.76E | 0 | 2012-2016 | 302258 | 210084.50 | 159.9 | 1.45 | 18042.91 | ＜0.001 |
| 10% | 10^th^ secondary cluster | Sichuan | 2 | 30.28N, 102.90E | 471.25 | 2016 | 157438 | 125821.13 | 139.0 | 1.25 | 3706.90 | ＜0.001 |
| 10% | 11^th^ secondary cluster | Shanxi | 3 | 37.70N, 112.38E | 424.93 | 2009 | 175728 | 143776.22 | 135.8 | 1.22 | 3344.71 | ＜0.001 |
| 10% | 12^th^ secondary cluster | Henan | 1 | 33.80N, 113.59E | 0 | 2014 | 129087 | 104819.13 | 136.9 | 1.23 | 2632.67 | ＜0.001 |


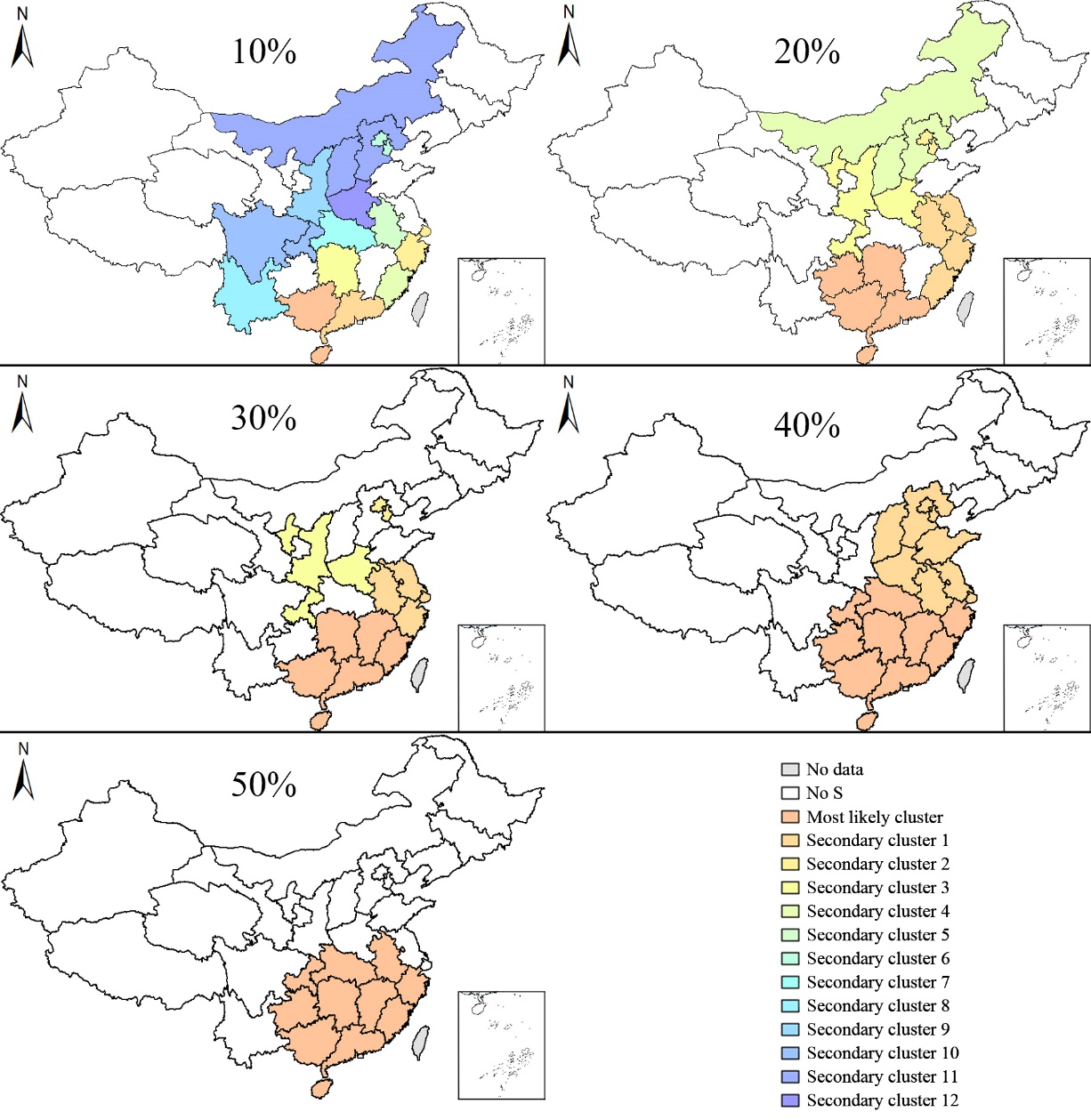


Table 3 Space-time analysis of OIDD

| Maximum Spatial Cluster Size | Cluster type | Location center | Cluster areas | Coordinates | Radius(Km) | Time(Year) | Number of Cases | Expected Cases | Annual Cases/10000 | Relative Risk | LLR | p-Value |
| --- | --- | --- | --- | --- | --- | --- | --- | --- | --- | --- | --- | --- |
| 50% | Most likely cluster | Beijing | 2 | 40.22N, 116.44E | 135.55 | 2006-2010 | 499320 | 90604.72 | 343.6 | 5.77 | 452831.47 | ＜0.001 |
| 50% | Secondary cluster | Fujian | 5 | 26.00N, 118.02E | 672.79 | 2012-2016 | 1845235 | 958099.52 | 120.1 | 2.16 | 371972.73 | ＜0.001 |
| 50% | 2^nd^ secondary cluster | Guizhou | 3 | 26.67N, 106.61E | 444.67 | 2015-2016 | 224173 | 142078.48 | 98.4 | 1.59 | 20511.25 | ＜0.001 |
| 50% | 3^rd^ secondary cluster | Xinjiang | 1 | 42.00N, 85.66E | 0 | 2006-2009 | 73852 | 52610.52 | 87.5 | 1.41 | 3829.96 | ＜0.001 |
| 50% | 4^th^ secondary cluster | Qinghai | 1 | 35.72N, 96.48E | 0 | 2012-2016 | 20305 | 18177.75 | 69.6 | 1.12 | 120.13 | ＜0.001 |
| 40% | Most likely cluster | Beijing | 2 | 40.22N, 116.44E | 135.55 | 2006-2010 | 499320 | 90604.72 | 343.6 | 5.77 | 452831.47 | ＜0.001 |
| 40% | Secondary cluster | Fujian | 5 | 26.00N, 118.02E | 672.79 | 2012-2016 | 1845235 | 958099.52 | 120.1 | 2.16 | 371972.73 | ＜0.001 |
| 40% | 2^nd^ secondary cluster | Guizhou | 3 | 26.67N, 106.61E | 444.67 | 2015-2016 | 224173 | 142078.48 | 98.4 | 1.59 | 20511.25 | ＜0.001 |
| 40% | 3^rd^ secondary cluster | Xinjiang | 1 | 42.00N, 85.66E | 0 | 2006-2009 | 73852 | 52610.52 | 87.5 | 1.41 | 3829.96 | ＜0.001 |
| 40% | 4^th^ secondary cluster | Gansu | 2 | 35.95N, 103.80E | 250.73 | 2016 | 23885 | 20509.38 | 72.6 | 1.17 | 264.32 | ＜0.001 |
| 40% | 5^th^ secondary cluster | Qinghai | 1 | 35.72N, 96.48E | 0 | 2012-2016 | 20305 | 18177.75 | 69.6 | 1.12 | 120.13 | ＜0.001 |
| 30% | Most likely cluster | Beijing | 2 | 40.22N, 116.44E | 135.55 | 2006-2010 | 499320 | 90604.72 | 343.6 | 5.77 | 452831.47 | ＜0.001 |
| 30% | Secondary cluster | Fujian | 5 | 26.00N, 118.02E | 672.79 | 2012-2016 | 1845235 | 958099.52 | 120.1 | 2.16 | 371972.73 | ＜0.001 |
| 30% | 2^nd^ secondary cluster | Guizhou | 3 | 26.67N, 106.61E | 444.67 | 2015-2016 | 224173 | 142078.48 | 98.4 | 1.59 | 20511.25 | ＜0.001 |
| 30% | 3^rd^ secondary cluster | Ningxia | 1 | 37.37N, 105.99E | 0 | 2012-2016 | 43808 | 20614.31 | 132.5 | 2.13 | 9859.54 | ＜0.001 |
| 30% | 4^th^ secondary cluster | Xinjiang | 1 | 42.00N, 85.66E | 0 | 2006-2009 | 73852 | 52610.52 | 87.5 | 1.41 | 3829.96 | ＜0.001 |
| 30% | 5^th^ secondary cluster | Qinghai | 1 | 35.72N, 96.48E | 0 | 2012-2016 | 20305 | 18177.75 | 69.6 | 1.12 | 120.13 | ＜0.001 |
| 20% | Most likely cluster | Beijing | 2 | 40.22N, 116.44E | 135.55 | 2006-2010 | 499320 | 90604.72 | 343.6 | 5.77 | 452831.47 | ＜0.001 |
| 20% | Secondary cluster | Fujian | 4 | 26.00N, 118.02E | 560.26 | 2011-2015 | 1473805 | 762558.94 | 120.5 | 2.11 | 290835.61 | ＜0.001 |
| 20% | 2^nd^ secondary cluster | Anhui | 1 | 32.01N, 117.19E | 0 | 2012-2016 | 383340 | 189853.04 | 125.9 | 2.06 | 77974.09 | ＜0.001 |
| 20% | 3^rd^ secondary cluster | Guizhou | 3 | 26.67N, 106.61E | 444.67 | 2015-2016 | 224173 | 142078.48 | 98.4 | 1.59 | 20511.25 | ＜0.001 |
| 20% | 4^th^ secondary cluster | Ningxia | 1 | 37.37N, 105.99E | 0 | 2012-2016 | 43808 | 20614.31 | 132.5 | 2.13 | 9859.54 | ＜0.001 |
| 20% | 5^th^ secondary cluster | Xinjiang | 1 | 42.00N, 85.66E | 0 | 2006-2009 | 73852 | 52610.52 | 87.5 | 1.41 | 3829.96 | ＜0.001 |
| 20% | 6^th^ secondary cluster | Hubei | 1 | 30.90N, 113.03E | 0 | 2016 | 42472 | 36740.10 | 72.1 | 1.16 | 427.33 | ＜0.001 |
| 20% | 7^th^ secondary cluster | Qinghai | 1 | 35.72N, 96.48E | 0 | 2012-2016 | 20305 | 18177.75 | 69.6 | 1.12 | 120.13 | ＜0.001 |
| 10% | Most likely cluster | Beijing | 2 | 40.22N, 116.44E | 135.55 | 2006-2010 | 499320 | 90604.72 | 343.6 | 5.77 | 452831.47 | ＜0.001 |
| 10% | Secondary cluster | Zhejiang | 1 | 29.10N, 120.10E | 0 | 2006-2010 | 546671 | 161369.57 | 211.2 | 3.54 | 290075.66 | ＜0.001 |
| 10% | 2^nd^ secondary cluster | Guangdong | 1 | 23.28N, 113.36E | 0 | 2010-2014 | 653672 | 329679.14 | 123.6 | 2.06 | 129444.93 | ＜0.001 |
| 10% | 3^rd^ secondary cluster | Anhui | 1 | 32.01N, 117.19E | 0 | 2012-2016 | 383340 | 189853.04 | 125.9 | 2.06 | 77974.09 | ＜0.001 |
| 10% | 4^th^ secondary cluster | Guangxi | 1 | 23.02N, 108.41E | 0 | 2015-2016 | 138363 | 60073.54 | 143.6 | 2.32 | 37485.58 | ＜0.001 |
| 10% | 5^th^ secondary cluster | Chongqing | 1 | 29.80N, 107.77E | 0 | 2012-2016 | 161046 | 93357.95 | 107.5 | 1.74 | 20375.04 | ＜0.001 |
| 10% | 6^th^ secondary cluster | Ningxia | 1 | 37.37N, 105.99E | 0 | 2012-2016 | 43808 | 20614.31 | 132.5 | 2.13 | 9859.54 | ＜0.001 |
| 10% | 7^th^ secondary cluster | Xinjiang | 1 | 42.00N, 85.69E | 0 | 2006-2009 | 73852 | 52610.52 | 87.5 | 1.41 | 3829.96 | ＜0.001 |
| 10% | 8^th^ secondary cluster | Fujian | 2 | 26.00N, 118.02E | 305.25 | 2006-2009 | 149848 | 199231.07 | 46.9 | 0.75 | 6835.41 | ＜0.001 |
| 10% | 9^th^ secondary cluster | Hubei | 1 | 30.90N, 113.03E | 0 | 2016 | 42472 | 36740.10 | 72.1 | 1.16 | 427.33 | ＜0.001 |
| 10% | 10^th^ secondary cluster | Shanxi | 2 | 37.70N, 112.38E | 0 | 2013-2105 | 218325 | 206126.17 | 66.0 | 1.06 | 362.36 | ＜0.001 |
| 10% | 11^th^ secondary cluster | Qinghai | 1 | 35.72N, 96.48E | 0 | 2012-2016 | 20305 | 18177.75 | 69.6 | 1.12 | 120.13 | ＜0.001 |
| 10% | 12^th^ secondary cluster | Hunan | 1 | 28.02N, 111.58E | 0 | 2016 | 60647 | 59509.47 | 63.5 | 1.02 | 10.87 | ＜0.001 |


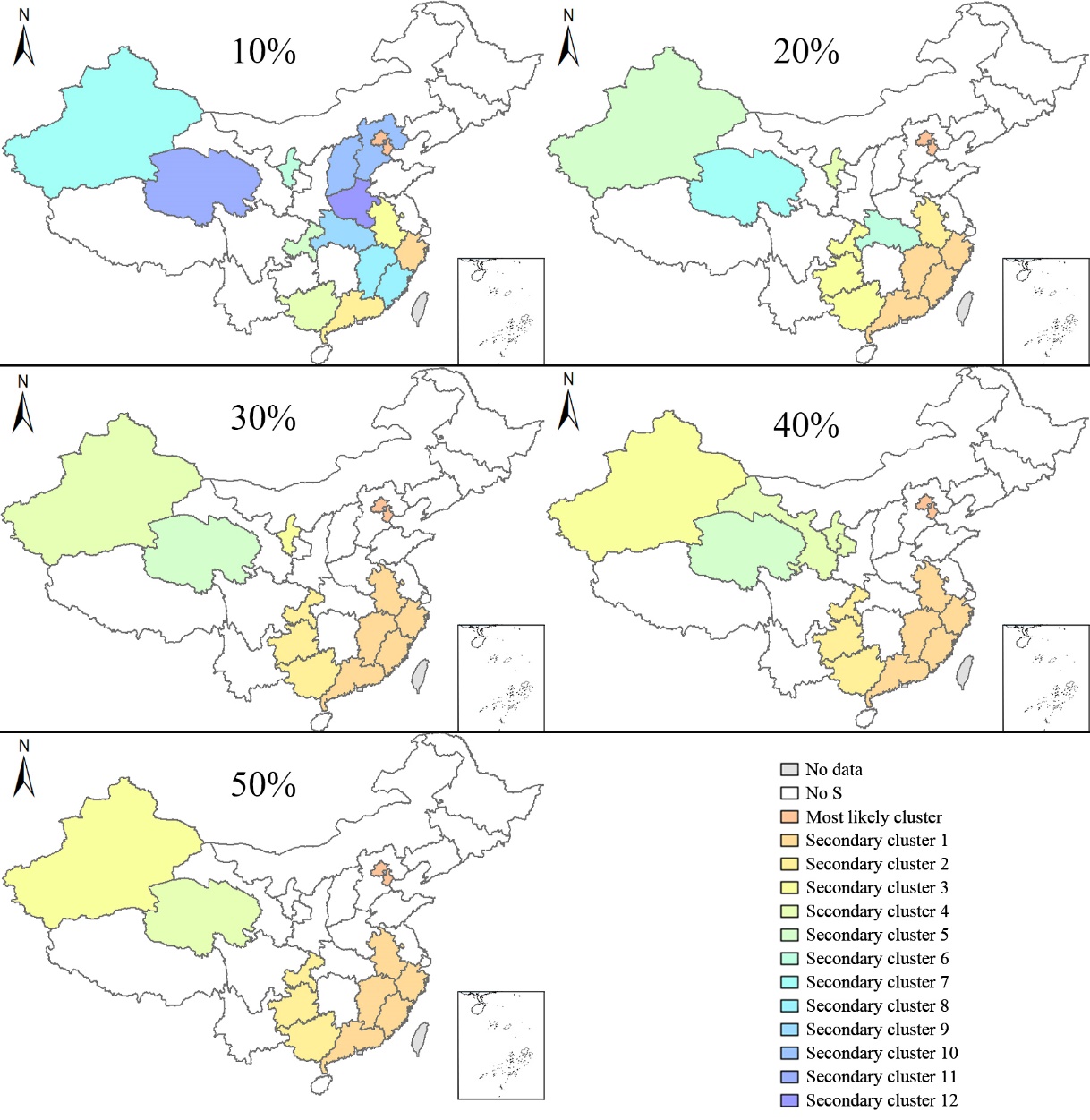


Table 4 Space-time analysis of TAP

| Maximum Spatial Cluster Size | Cluster type | Location center | Cluster areas | Coordinates | Radius(Km) | Time(Year) | Number of Cases | Expected Cases | Annual Cases/10000 | Relative Risk | LLR | p-Value |
| --- | --- | --- | --- | --- | --- | --- | --- | --- | --- | --- | --- | --- |
| 50% | Most likely cluster | Yunnan | 2 | 24.14N, 101.30E | 602.35 | 2006-2010 | 39400 | 4665.15 | 9.6 | 10.74 | 53337.37 | ＜0.001 |
| 50% | Secondary cluster | Zhejiang | 1 | 29.10N, 120.10E | 0 | 2006-2008 | 6297 | 1726.13 | 4.1 | 3.75 | 3642.35 | ＜0.001 |
| 50% | 2^nd^ secondary cluster | Shanxi | 1 | 37.70N, 112.38E | 0 | 2014 | 837 | 414.40 | 2.3 | 2.02 | 166.34 | ＜0.001 |
| 40% | Most likely cluster | Yunnan | 2 | 24.14N, 101.30E | 602.35 | 2006-2010 | 39400 | 4665.15 | 9.6 | 10.74 | 53337.37 | ＜0.001 |
| 40% | Secondary cluster | Zhejiang | 1 | 29.10N, 120.10E | 0 | 2006-2008 | 6297 | 1726.13 | 4.1 | 3.75 | 3642.35 | ＜0.001 |
| 40% | 2^nd^ secondary cluster | Shanxi | 1 | 37.70N, 112.38E | 0 | 2014 | 837 | 414.40 | 2.3 | 2.02 | 166.34 | ＜0.001 |
| 30% | Most likely cluster | Yunnan | 2 | 24.14N, 101.30E | 602.35 | 2006-2010 | 39400 | 4665.15 | 9.6 | 10.74 | 53337.37 | ＜0.001 |
| 30% | Secondary cluster | Zhejiang | 1 | 29.10N, 120.10E | 0 | 2006-2008 | 6297 | 1726.13 | 4.1 | 3.75 | 3642.35 | ＜0.001 |
| 30% | 2^nd^ secondary cluster | Shanxi | 1 | 37.70N, 112.38E | 0 | 2014 | 837 | 414.40 | 2.3 | 2.02 | 166.34 | ＜0.001 |
| 20% | Most likely cluster | Yunnan | 2 | 24.14N, 101.30E | 602.35 | 2006-2010 | 39400 | 4665.15 | 9.6 | 10.74 | 53337.37 | ＜0.001 |
| 20% | Secondary cluster | Zhejiang | 1 | 29.10N, 120.10E | 0 | 2006-2008 | 6297 | 1726.13 | 4.1 | 3.75 | 3642.35 | ＜0.001 |
| 20% | 2^nd^ secondary cluster | Guangdong | 4 | 23.28N, 113.36E | 555.87 | 2006-2010 | 23244 | 14403.13 | 1.8 | 1.71 | 2544.47 | ＜0.001 |
| 20% | 3^rd^ secondary cluster | Xinjiang | 1 | 42.00N, 85.66E | 0 | 2006-2010 | 3785 | 1207.51 | 3.6 | 3.18 | 1766.92 | ＜0.001 |
| 20% | 4^th^ secondary cluster | Shanxi | 1 | 37.70N, 112.38E | 0 | 2014 | 837 | 414.40 | 2.3 | 2.02 | 166.34 | ＜0.001 |
| 10% | Most likely cluster | Yunnan | 2 | 24.14N, 101.30E | 602.35 | 2006-2010 | 39400 | 4665.15 | 9.6 | 10.74 | 53337.37 | ＜0.001 |
| 10% | Secondary cluster | Zhejiang | 1 | 29.10N, 120.10E | 0 | 2006-2008 | 6297 | 1726.13 | 4.1 | 3.75 | 3642.35 | ＜0.001 |
| 10% | 2^nd^ secondary cluster | Xinjiang | 1 | 42.00N, 85.66E | 0 | 2006-2010 | 3785 | 1207.51 | 3.6 | 3.18 | 1766.92 | ＜0.001 |
| 10% | 3^rd^ secondary cluster | Hainan | 2 | 19.22N, 109.77E | 444.53 | 2006-2010 | 6490 | 3187.44 | 2.3 | 2.08 | 1345.58 | ＜0.001 |
| 10% | 4^th^ secondary cluster | Hunan | 1 | 28.01N, 111.58E | 0 | 2006-2009 | 5167 | 2899.33 | 2.0 | 1.81 | 733.60 | ＜0.001 |
| 10% | 5^th^ secondary cluster | Guangdong | 1 | 23.27N, 113.35E | 0 | 2006-2010 | 8272 | 5555.98 | 1.7 | 1.51 | 599.19 | ＜0.001 |
| 10% | 6^th^ secondary cluster | Shanxi | 1 | 37.70N, 112.38E | 0 | 2014 | 837 | 414.40 | 2.3 | 2.02 | 166.34 | ＜0.001 |
| 10% | 7^th^ secondary cluster | Fujian | 1 | 26.00N, 118.02E | 0 | 2016 | 706 | 440.81 | 1.8 | 1.60 | 67.55 | ＜0.001 |


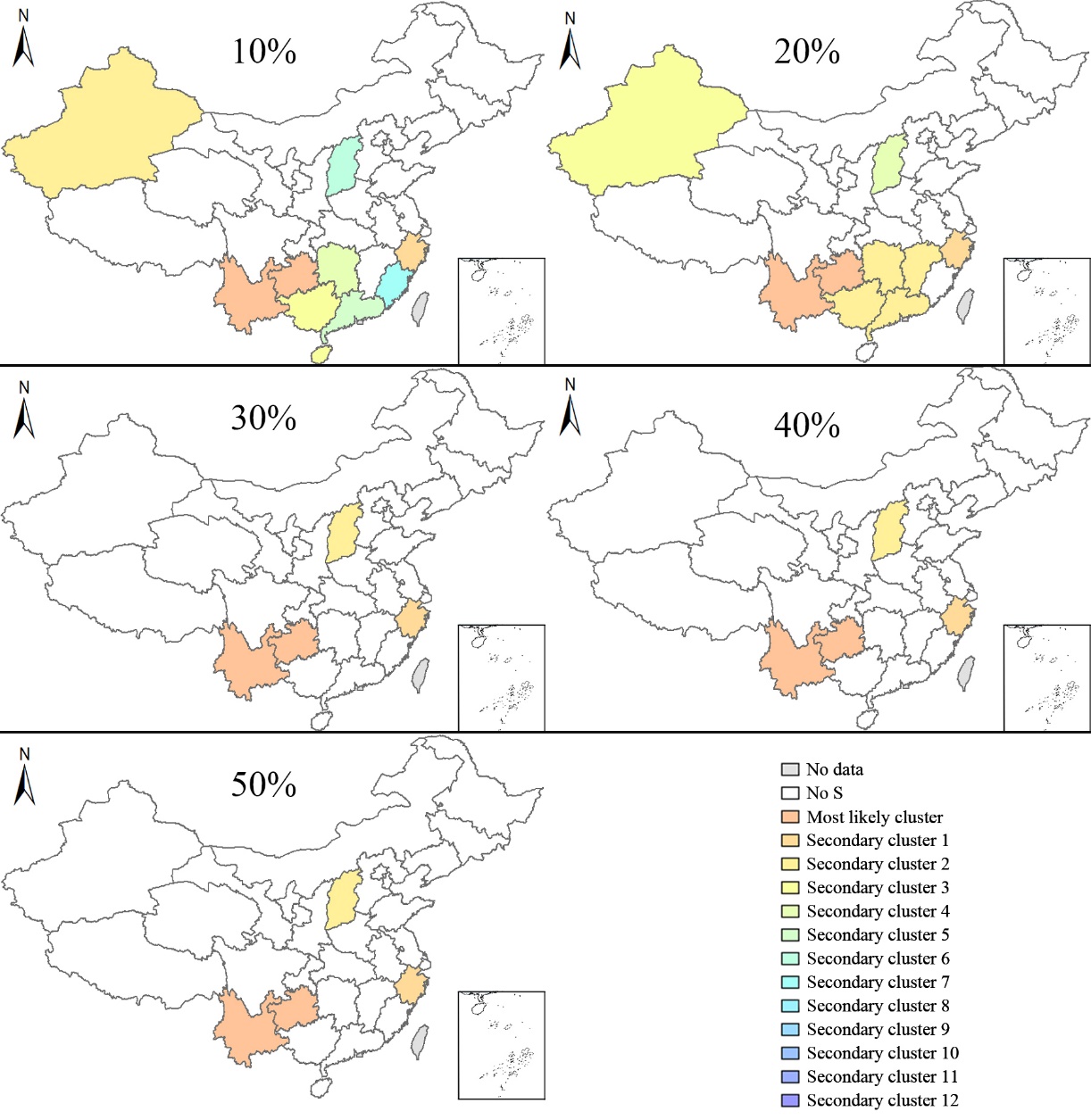


Table 5 Space-time analysis of dysentery

| Maximum Spatial Cluster Size | Cluster type | Location center | Cluster areas | Coordinates | Radius(Km) | Time(Year) | Number of Cases | Expected Cases | Annual Cases/10000 | Relative Risk | LLR | p-Value |
| --- | --- | --- | --- | --- | --- | --- | --- | --- | --- | --- | --- | --- |
| 50% | Most likely cluster | Xinjiang | 17 | 42.00N, 85.66E | 2723.61 | 2006-2010 | 1053358 | 545351.07 | 35.1 | 2.54 | 251530.50 | ＜0.001 |
| 50% | Secondary cluster | Zhejiang | 1 | 29.10N, 120.10E | 0 | 2006-2008 | 48455 | 27597.65 | 31.9 | 1.77 | 6500.61 | ＜0.001 |
| 40% | Most likely cluster | Beijing | 2 | 40.22N, 116.44E | 135.55 | 2006-2010 | 206127 | 26414.00 | 141.8 | 8.37 | 250036.55 | ＜0.001 |
| 40% | Secondary cluster | Qinghai | 10 | 35.72N, 96.48E | 1391.35 | 2006-2010 | 517262 | 264476.70 | 35.5 | 2.18 | 107939.77 | ＜0.001 |
| 40% | 2^nd^ secondary cluster | Anhui | 5 | 32.01N, 117.19E | 426.92 | 2006-2007 | 168754 | 122808.41 | 25.0 | 1.40 | 8103.40 | ＜0.001 |
| 30% | Most likely cluster | Beijing | 2 | 40.22N, 116.44E | 135.55 | 2006-2010 | 206127 | 26414.00 | 141.8 | 8.37 | 250036.55 | ＜0.001 |
| 30% | Secondary cluster | Qinghai | 10 | 35.72N, 96.48E | 1391.35 | 2006-2010 | 517262 | 264476.70 | 35.5 | 2.18 | 107939.77 | ＜0.001 |
| 30% | 2^nd^ secondary cluster | Anhui | 5 | 32.01N, 117.19E | 426.92 | 2006-2007 | 168754 | 122808.41 | 25.0 | 1.40 | 8103.40 | ＜0.001 |
| 20% | Most likely cluster | Beijing | 2 | 40.22N, 116.44E | 135.55 | 2006-2010 | 206127 | 26414.00 | 141.8 | 8.37 | 250036.55 | ＜0.001 |
| 20% | Secondary cluster | Qinghai | 8 | 35.72N, 96.48E | 1242.09 | 2006-2010 | 406122 | 189889.46 | 38.9 | 2.34 | 102201.42 | ＜0.001 |
| 20% | 2^nd^ secondary cluster | Guangxi | 3 | 23.02N, 108.41E | 444.67 | 2006-2007 | 62686 | 33962.06 | 33.5 | 1.87 | 9852.75 | ＜0.001 |
| 20% | 3^rd^ secondary cluster | Zhejiang | 1 | 29.10 N, 120.10E | 0 | 2006-2008 | 48455 | 27597.65 | 31.9 | 1.77 | 6500.61 | ＜0.001 |
| 20% | 4^th^ secondary cluster | Hubei | 2 | 30.90N, 113.03E | 326.52 | 2006-2008 | 111678 | 82340.82 | 24.7 | 1.37 | 4863.65 | ＜0.001 |
| 10% | Most likely cluster | Beijing | 2 | 40.22N, 116.44E | 135.55 | 2006-2010 | 206127 | 26414.00 | 141.8 | 8.37 | 250036.55 | ＜0.001 |
| 10% | Secondary cluster | Xinjiang | 5 | 42.00N, 85.66E | 1808.12 | 2006-2010 | 178515 | 56165.23 | 57.8 | 3.33 | 86984.82 | ＜0.001 |
| 10% | 2^nd^ secondary cluster | Chongqing | 2 | 29.80N, 107.77E | 365.86 | 2006-2010 | 116793 | 59130.08 | 35.9 | 2.02 | 22474.23 | ＜0.001 |
| 10% | 3^rd^ secondary cluster | Shanxi | 2 | 37.70N, 112.38E | 270.94 | 2006-2009 | 123212 | 75445.21 | 29.7 | 1.66 | 13110.35 | ＜0.001 |
| 10% | 4^th^ secondary cluster | Zhejiang | 1 | 29.10 N, 120.10E | 0 | 2006-2008 | 48455 | 27597.65 | 31.9 | 1.77 | 6500.61 | ＜0.001 |
| 10% | 5^th^ secondary cluster | Hubei | 1 | 30.90N, 113.03E | 0 | 2006-2009 | 59146 | 41484.09 | 25.9 | 1.44 | 3376.49 | ＜0.001 |
| 10% | 6^th^ secondary cluster | Henan | 1 | 33.80N, 113.59E | 0 | 2007-2007 | 47103 | 34075.10 | 25.1 | 1.39 | 2254.82 | ＜0.001 |
| 10% | 7^th^ secondary cluster | Hainan | 2 | 19.22N, 109.77E | 444.53 | 2006-2007 | 29520 | 20298.11 | 26.4 | 1.46 | 1850.58 | ＜0.001 |
| 10% | 8^th^ secondary cluster | Jiangxi | 1 | 27.73N, 115.63E | 0 | 2006-2008 | 33036 | 23836.04 | 25.2 | 1.39 | 1598.95 | ＜0.001 |
| 10% | 9^th^ secondary cluster | Anhui | 1 | 32.01N, 117.19E | 0 | 2011-2012 | 29336 | 21752.94 | 24.5 | 1.35 | 1201.20 | ＜0.001 |
| 10% | 10^th^ secondary cluster | Jiangsu | 2 | 32.47N, 119.97E | 213.91 | 2006 | 20407 | 17036.98 | 21.8 | 1.20 | 315.41 | ＜0.001 |


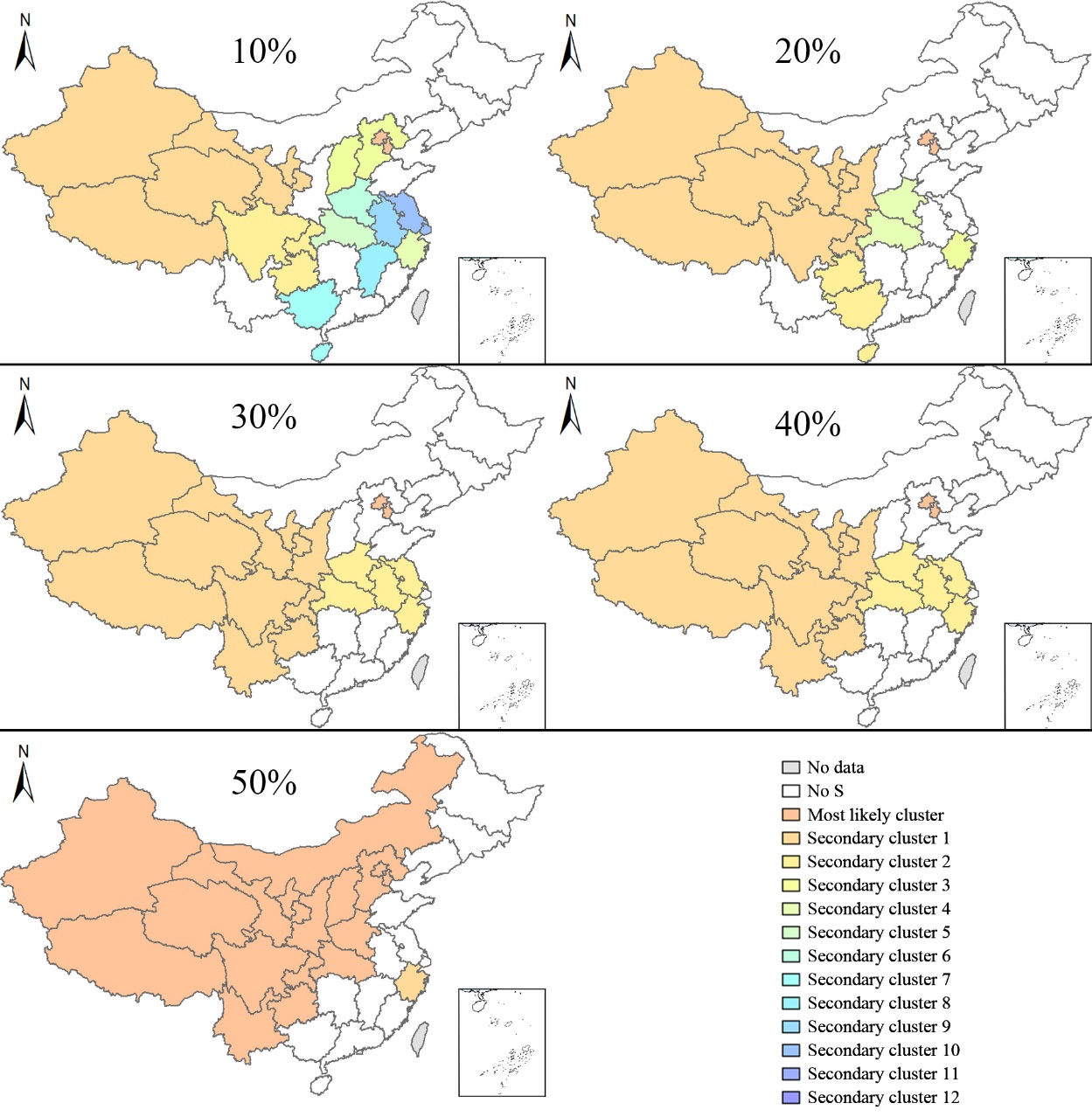


Table 6 Space-time analysis of hepatitis A

| Maximum Spatial Cluster Size | Cluster type | Location center | Cluster areas | Coordinates | Radius(Km) | Time(Year) | Number of Cases | Expected Cases | Annual Cases/10000 | Relative Risk | LLR | p-Value |
| --- | --- | --- | --- | --- | --- | --- | --- | --- | --- | --- | --- | --- |
| 50% | Most likely cluster | Xinjiang | 9 | 31.10N, 89.12E | 1790.28 | 2006-2010 | 142703 | 36975.74 | 11.3 | 5.28 | 102737.42 | ＜0.001 |
| 50% | Secondary cluster | Henan | 2 | 33.80N, 113.59E | 326.52 | 2006-2009 | 29048 | 17636.02 | 4.8 | 1.69 | 3242.92 | ＜0.001 |
| 50% | 2^nd^ secondary cluster | Liaoning | 1 | 41.47N, 123.52E | 0 | 2015-2016 | 4919 | 2556.40 | 5.6 | 1.93 | 863.46 | ＜0.001 |
| 40% | Most likely cluster | Xinjiang | 9 | 31.10N, 89.12E | 1790.28 | 2006-2010 | 142703 | 36975.74 | 11.3 | 5.28 | 102737.42 | ＜0.001 |
| 40% | Secondary cluster | Henan | 2 | 33.80N, 113.59E | 326.52 | 2006-2009 | 29048 | 17636.02 | 4.8 | 1.69 | 3242.92 | ＜0.001 |
| 40% | 2^nd^ secondary cluster | Fujian | 3 | 26.00N, 118.02E | 400.88 | 2006-2007 | 12908 | 7547.36 | 5.0 | 1.73 | 1600.66 | ＜0.001 |
| 40% | 3^rd^ secondary cluster | Liaoning | 1 | 41.47N, 123.52E | 0 | 2015-2016 | 4919 | 2556.40 | 5.6 | 1.93 | 863.46 | ＜0.001 |
| 30% | Most likely cluster | Xinjiang | 9 | 31.10N, 89.12E | 1790.28 | 2006-2010 | 142703 | 36975.74 | 11.3 | 5.28 | 102737.42 | ＜0.001 |
| 30% | Secondary cluster | Henan | 2 | 33.80N, 113.59E | 326.52 | 2006-2009 | 29048 | 17636.02 | 4.8 | 1.69 | 3242.92 | ＜0.001 |
| 30% | 2^nd^ secondary cluster | Fujian | 3 | 26.00N, 118.02E | 400.88 | 2006-2007 | 12908 | 7547.36 | 5.0 | 1.73 | 1600.66 | ＜0.001 |
| 30% | 3^rd^ secondary cluster | Liaoning | 1 | 41.47N, 123.52E | 0 | 2015-2016 | 4919 | 2556.40 | 5.6 | 1.93 | 863.46 | ＜0.001 |
| 20% | Most likely cluster | Xizang | 9 | 31.10N, 89.12E | 1790.28 | 2006-2010 | 142703 | 36975.74 | 11.3 | 5.28 | 102737.42 | ＜0.001 |
| 20% | Secondary cluster | Henan | 2 | 33.80N, 113.59E | 326.52 | 2006-2009 | 29048 | 17636.02 | 4.8 | 1.69 | 3242.92 | ＜0.001 |
| 20% | 2^nd^ secondary cluster | Fujian | 3 | 26.00N, 118.02E | 400.88 | 2006-2007 | 12908 | 7547.36 | 5.0 | 1.73 | 1600.66 | ＜0.001 |
| 20% | 3^rd^ secondary cluster | Liaoning | 1 | 41.47N, 123.52E | 0 | 2015-2016 | 4919 | 2556.40 | 5.6 | 1.93 | 863.46 | ＜0.001 |
| 10% | Most likely cluster | Xinjiang | 5 | 42.00N, 85.66E | 1808.12 | 2006-2010 | 178515 | 56165.23 | 57.8 | 3.33 | 86984.82 | ＜0.001 |
| 10% | Secondary cluster | Yunnan | 2 | 24.14N, 101.30E | 602.35 | 2006-2010 | 50005 | 11966.06 | 12.2 | 4.60 | 35260.56 | ＜0.001 |
| 10% | 2^nd^ secondary cluster | Sichuan | 2 | 30.28N, 102.90E | 471.25 | 2006-2010 | 38483 | 15999.07 | 7.0 | 2.54 | 11915.42 | ＜0.001 |
| 10% | 3^rd^ secondary cluster | Henan | 1 | 33.80N, 113.59E | 0 | 2007-2007 | 47103 | 34075.10 | 25.1 | 1.39 | 2254.82 | ＜0.001 |
| 10% | 4^th^ secondary cluster | Jiangxi | 1 | 27.73N, 115.63E | 0 | 2006-2008 | 33036 | 23836.04 | 25.2 | 1.39 | 1598.95 | ＜0.001 |
| 10% | 5^th^ secondary cluster | Liaoning | 1 | 41.47N, 123.52E | 0 | 2015-2016 | 4919 | 2556.40 | 5.6 | 1.93 | 863.46 | ＜0.001 |
| 10% | 6^th^ secondary cluster | Hunan | 2 | 28.02N, 111.58E | 349.86 | 2006-2007 | 9856 | 7020.72 | 4.1 | 1.41 | 517.57 | ＜0.001 |
| 10% | 7^th^ secondary cluster | Hainan | 2 | 19.22N, 109.77E | 444.53 | 2006-2007 | 29520 | 20298.11 | 26.4 | 1.46 | 1850.58 | ＜0.001 |
| 10% | 8^th^ secondary cluster | Zhejiang | 1 | 29.10N, 120.10E | 0 | 2006-2007 | 4432 | 2927.88 | 4.4 | 1.52 | 335.92 | ＜0.001 |
| 10% | 9^th^ secondary cluster | Neimenggu | 1 | 41.39N, 111.07E | 0 | 2007 | 1053 | 700.83 | 4.4 | 1.50 | 76.69 | ＜0.001 |
| 10% | 10^th^ secondary cluster | Anhui | 1 | 32.01N, 117.19E | 0 | 2006-2007 | 3867 | 3563.77 | 3.2 | 1.09 | 12.66 | ＜0.001 |
| 10% | 11^th^ secondary cluster | Shanxi | 1 | 37.70N, 112.38E | 0 | 2014 | 1229 | 1062.94 | 3.4 | 1.16 | 12.38 | ＜0.001 |
| 10% | 12^th^ secondary cluster | Jiangsu | 1 | 32.47N, 119.97E | 0 | 2006-2007 | 4673 | 4423.89 | 3.1 | 1.06 | 6.96 | ＜0.001 |


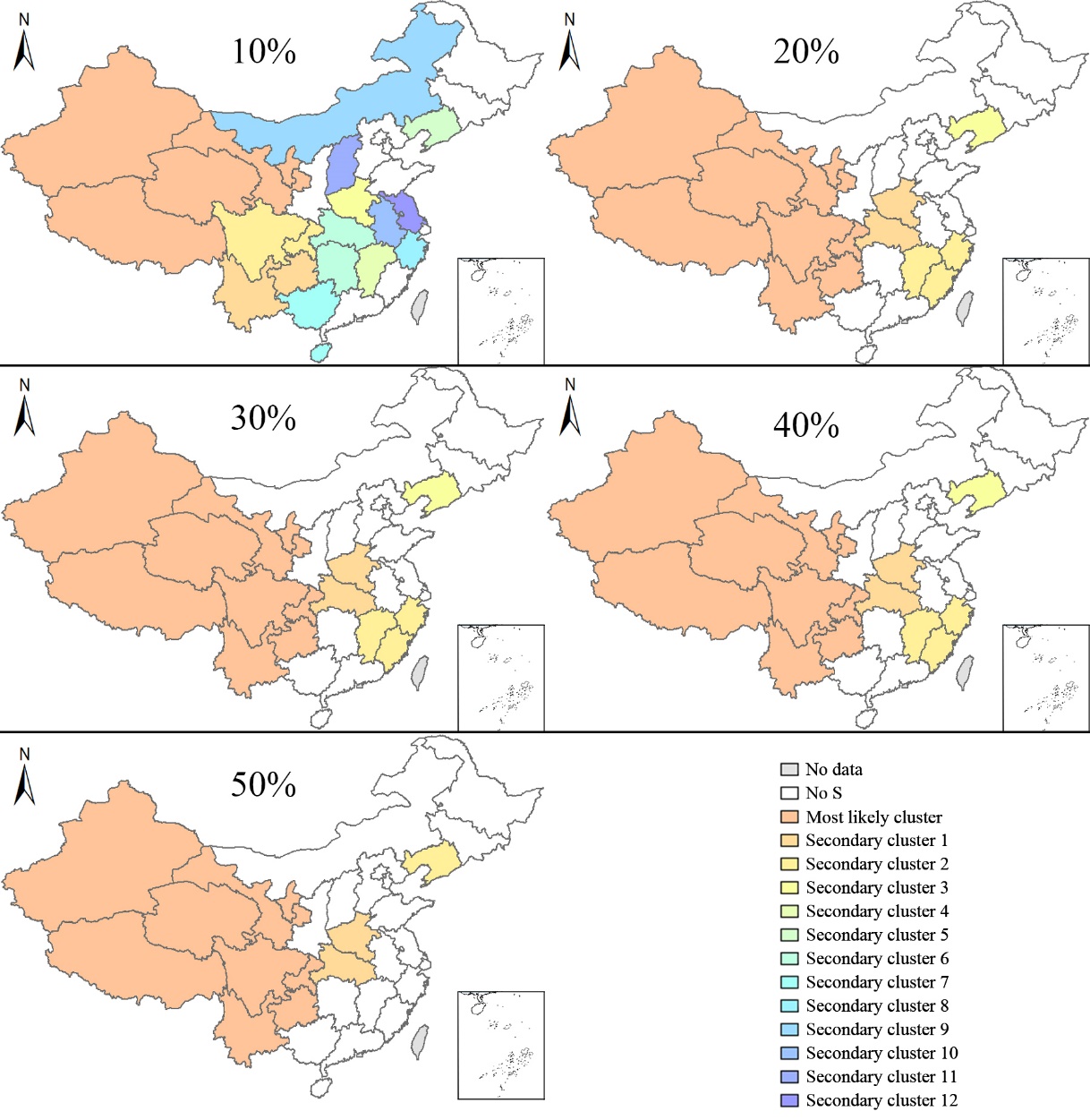


Table 6 Space-time analysis of hepatitis E

| Maximum Spatial Cluster Size | Cluster type | Location center | Cluster areas | Coordinates | Radius(Km) | Time(Year) | Number of Cases | Expected Cases | Annual Cases/10000 | Relative Risk | LLR | p-Value |
| --- | --- | --- | --- | --- | --- | --- | --- | --- | --- | --- | --- | --- |
| 50% | Most likely cluster | Jiangxi | 8 | 27.73N, 115.63E | 671.06 | 2010-2014 | 76951 | 46212.61 | 3.0 | 1.93 | 10730.60 | ＜0.001 |
| 50% | Secondary cluster | Liaoning | 1 | 41.47N, 123.52E | 0 | 2006-2010 | 8095 | 3941.52 | 3.7 | 2.09 | 1705.11 | ＜0.001 |
| 50% | 2^nd^ secondary cluster | Chongqing | 1 | 29.80N, 107.77E | 0 | 2013-2016 | 3485 | 2194.17 | 2.9 | 1.60 | 324.69 | ＜0.001 |
| 50% | 3^rd^ secondary cluster | Xinjiang | 1 | 42.00N, 85.66E | 0 | 2015 | 522 | 429.88 | 2.2 | 1.21 | 9.25 | ＜0.001 |
| 40% | Most likely cluster | Jiangxi | 8 | 27.73N, 115.63E | 671.06 | 2010-2014 | 76951 | 46212.61 | 3.0 | 1.93 | 10730.60 | ＜0.001 |
| 40% | Secondary cluster | Liaoning | 1 | 41.47N, 123.52E | 0 | 2006-2010 | 8095 | 3941.52 | 3.7 | 2.09 | 1705.11 | ＜0.001 |
| 40% | 2^nd^ secondary cluster | Beijing | 2 | 40.22N, 116.44E | 135.55 | 2006-2007 | 1903 | 988.43 | 3.5 | 1.93 | 333.59 | ＜0.001 |
| 40% | 3^rd^ secondary cluster | Chongqing | 1 | 29.80N, 107.77E | 0 | 2013-2016 | 3485 | 2194.17 | 2.9 | 1.60 | 324.69 | ＜0.001 |
| 40% | 4^th^ secondary cluster | Xinjiang | 1 | 42.00N, 85.66E | 0 | 2015 | 522 | 429.88 | 2.2 | 1.21 | 9.25 | ＜0.001 |
| 30% | Most likely cluster | Zhejiang | 7 | 29.10N, 120.10E | 709.15 | 2010-2014 | 59410 | 32664.82 | 3.3 | 2.05 | 10368.95 | ＜0.001 |
| 30% | Secondary cluster | Hainan | 3 | 19.22N, 109.77E | 583.68 | 2012-2016 | 22737 | 14988.40 | 2.8 | 1.56 | 1845.93 | ＜0.001 |
| 30% | 2^nd^ secondary cluster | Liaoning | 1 | 41.47N, 123.52E | 0 | 2006-2010 | 8095 | 3941.52 | 3.7 | 2.09 | 1705.11 | ＜0.001 |
| 30% | 3^rd^ secondary cluster | Beijing | 2 | 40.22N, 116.44E | 135.55 | 2006-2007 | 1903 | 988.43 | 3.5 | 1.93 | 333.59 | ＜0.001 |
| 30% | 4^th^ secondary cluster | Chongqing | 1 | 29.80N, 107.77E | 0 | 2013-2016 | 3485 | 2194.17 | 2.9 | 1.60 | 324.69 | ＜0.001 |
| 30% | 5^th^ secondary cluster | Xinjiang | 1 | 42.00N, 85.66E | 0 | 2015 | 522 | 429.88 | 2.2 | 1.21 | 9.25 | ＜0.001 |
| 20% | Most likely cluster | Zhejiang | 5 | 29.10N, 120.10E | 426.92 | 2010-2014 | 46163 | 23285.66 | 3.6 | 2.19 | 9815.80 | ＜0.001 |
| 20% | Secondary cluster | Hubei | 1 | 30.90N, 113.03E | 0 | 2012-2016 | 11505 | 5318.00 | 3.9 | 2.22 | 2764.53 | ＜0.001 |
| 20% | 2^nd^ secondary cluster | Guangdong | 2 | 23.28N, 113.36E | 506.24 | 2012-2016 | 21756 | 14164.67 | 2.8 | 1.58 | 1859.44 | ＜0.001 |
| 20% | 3^rd^ secondary cluster | Liaoning | 1 | 41.47N, 123.52E | 0 | 2006-2010 | 8095 | 3941.52 | 3.7 | 2.09 | 1705.11 | ＜0.001 |
| 20% | 4^th^ secondary cluster | Beijing | 2 | 40.22N, 116.44E | 135.55 | 2006-2007 | 1903 | 988.43 | 3.5 | 1.93 | 333.59 | ＜0.001 |
| 20% | 5^th^ secondary cluster | Chongqing | 1 | 29.80N, 107.77E | 0 | 2013-2016 | 3485 | 2194.17 | 2.9 | 1.60 | 324.69 | ＜0.001 |
| 20% | 6^th^ secondary cluster | Xinjiang | 1 | 42.00N, 85.66E | 0 | 2015 | 522 | 429.88 | 2.2 | 1.21 | 9.25 | ＜0.001 |
| 10% | Most likely cluster | Jiangsu | 1 | 32.47N, 119.97E | 0 | 2010-2014 | 18568 | 7222.48 | 4.7 | 2.69 | 6437.10 | ＜0.001 |
| 10% | Secondary cluster | Hubei | 1 | 30.90N, 113.03E | 0 | 2012-2016 | 11505 | 5318.00 | 3.9 | 2.22 | 2764.53 | ＜0.001 |
| 10% | 2^nd^ secondary cluster | Zhejiang | 1 | 29.10N, 120.10E | 0 | 2007-2011 | 10607 | 4809.70 | 4.0 | 2.25 | 2655.75 | ＜0.001 |
| 10% | 3^rd^ secondary cluster | Liaoning | 1 | 41.47N, 123.52E | 0 | 2006-2010 | 8095 | 3941.52 | 3.7 | 2.09 | 1705.11 | ＜0.001 |
| 10% | 4^th^ secondary cluster | Guangdong | 1 | 23.28N, 113.36E | 0 | 2010-2014 | 14576 | 9650.15 | 2.8 | 1.54 | 1132.50 | ＜0.001 |
| 10% | 5^th^ secondary cluster | Anhui | 1 | 32.01N, 117.19 | 0 | 2010-2014 | 9132 | 5484.19 | 3.0 | 1.69 | 1034.15 | ＜0.001 |
| 10% | 6^th^ secondary cluster | Guangxi | 1 | 23.02N, 108.41E | 0 | 2012-2016 | 7369 | 4342.39 | 3.1 | 1.72 | 887.94 | ＜0.001 |
| 10% | 7^th^ secondary cluster | Fujian | 1 | 26.00N, 118.02E | 0 | 2011-2015 | 5456 | 3446.55 | 2.9 | 1.60 | 504.36 | ＜0.001 |
| 10% | 8^th^ secondary cluster | Beijing | 2 | 40.22N, 116.44E | 135.55 | 2006-2007 | 1903 | 988.43 | 3.5 | 1.93 | 333.59 | ＜0.001 |
| 10% | 9^th^ secondary cluster | Chongqing | 1 | 29.80N, 107.77E | 0 | 2013-2016 | 3485 | 2194.17 | 2.9 | 1.60 | 324.69 | ＜0.001 |
| 10% | 10^th^ secondary cluster | Yunnan | 1 | 24.14N, 101.30E | 0 | 2015-2016 | 2653 | 1736.64 | 2.8 | 1.53 | 209.39 | ＜0.001 |
| 10% | 11^th^ secondary cluster | Xinjiang | 1 | 42.00N, 85.66E | 0 | 2015 | 522 | 429.88 | 2.2 | 1.21 | 9.25 | ＜0.001 |


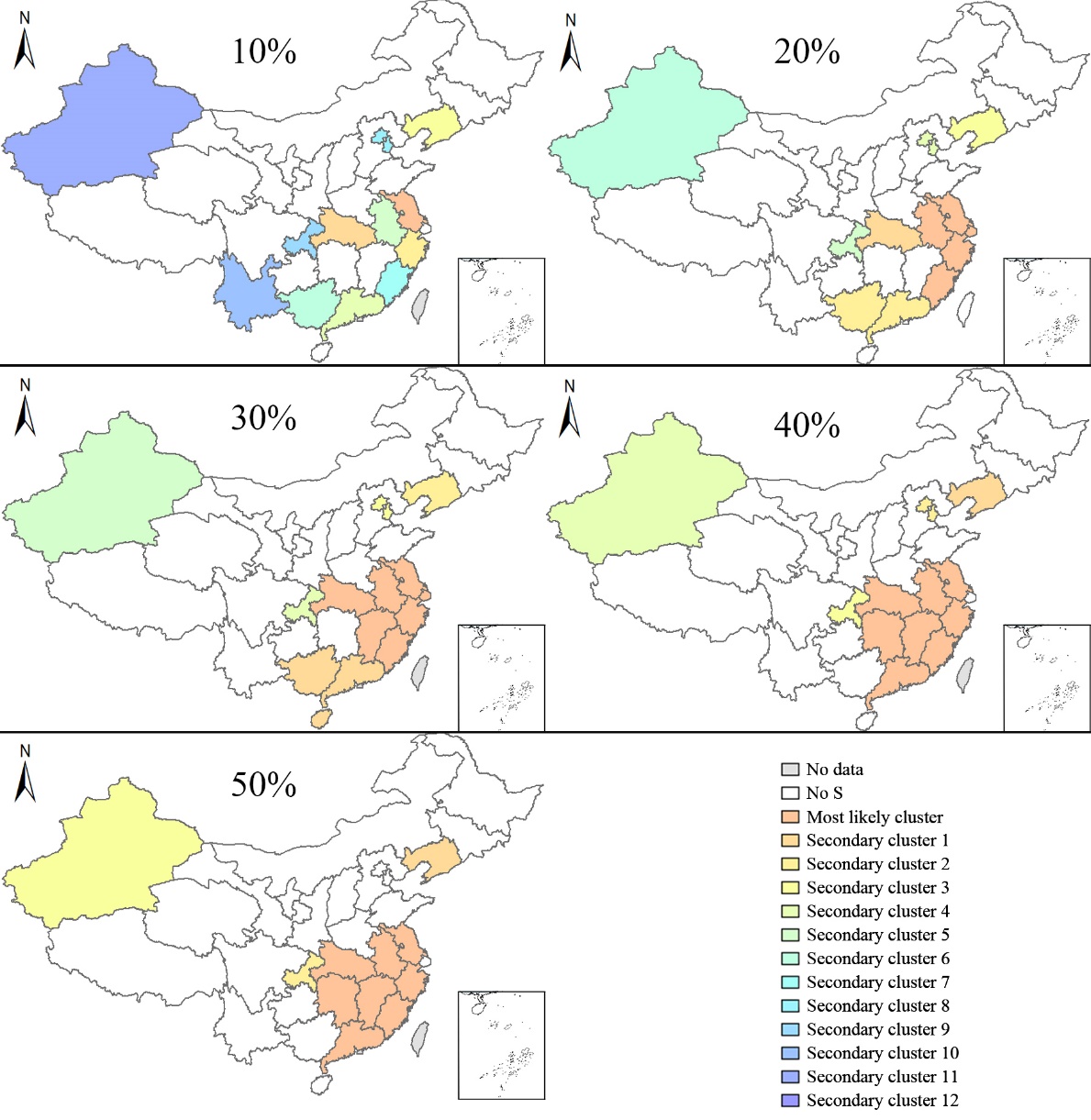


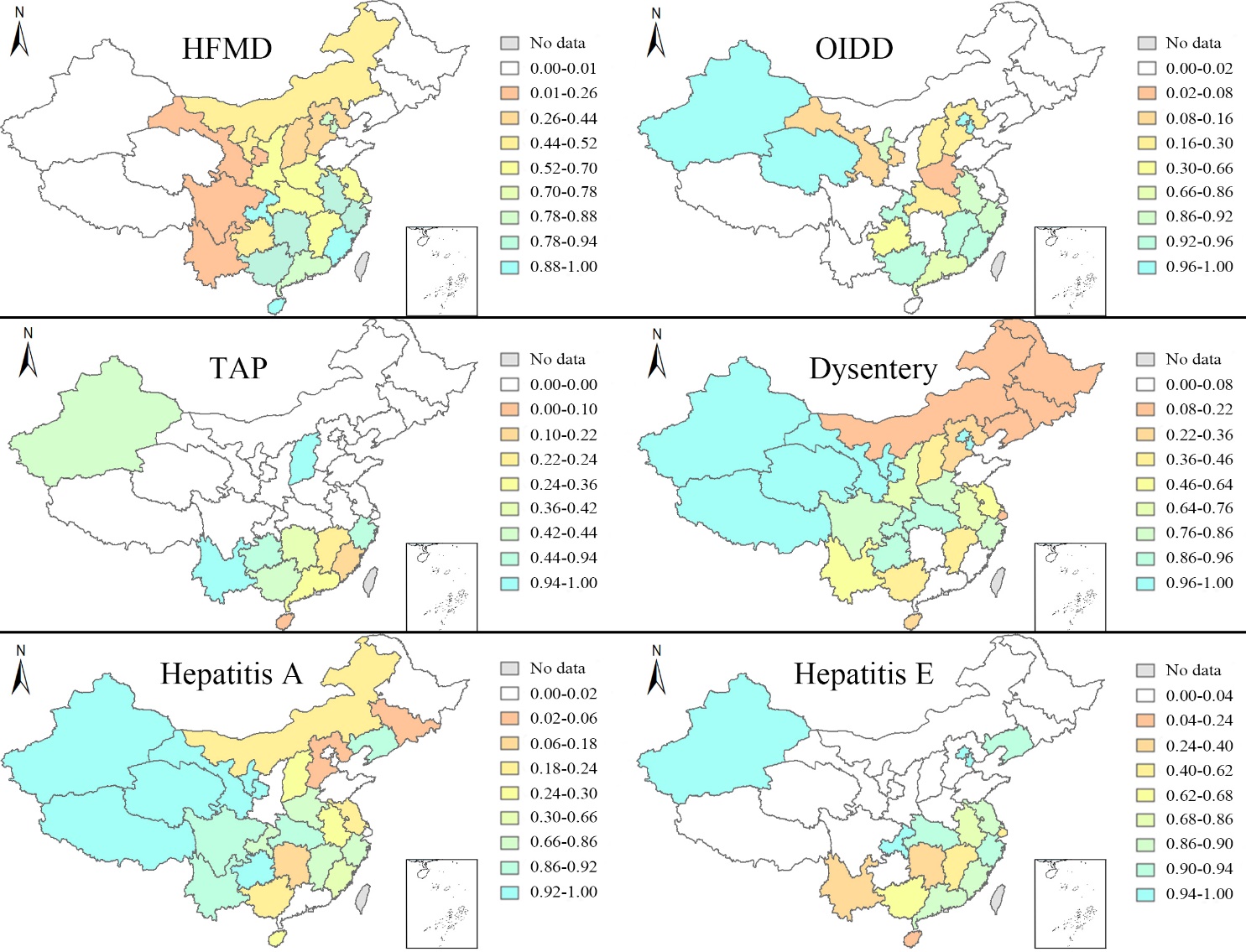


The Results of reliability among different province and diseases
